# Molecular Recognition of CCR5 by an HIV-1 gp120 V3 Loop

**DOI:** 10.1371/journal.pone.0095767

**Published:** 2014-04-24

**Authors:** Phanourios Tamamis, Christodoulos A. Floudas

**Affiliations:** Department of Chemical and Biological Engineering, Princeton University, Princeton, New Jersey, United States of America; University of Leeds, United Kingdom

## Abstract

The binding of protein HIV-1 gp120 to coreceptors CCR5 or CXCR4 is a key step of the HIV-1 entry to the host cell, and is predominantly mediated through the V3 loop fragment of HIV-1 gp120. In the present work, we delineate the molecular recognition of chemokine receptor CCR5 by a dual tropic HIV-1 gp120 V3 loop, using a comprehensive set of computational tools predominantly based on molecular dynamics simulations and free energy calculations. We report, what is to our knowledge, the first complete HIV-1 gp120 V3 loop : CCR5 complex structure, which includes the whole V3 loop and the N-terminus of CCR5, and exhibits exceptional agreement with previous experimental findings. The computationally derived structure sheds light into the functional role of HIV-1 gp120 V3 loop and CCR5 residues associated with the HIV-1 coreceptor activity, and provides insights into the HIV-1 coreceptor selectivity and the blocking mechanism of HIV-1 gp120 by maraviroc. By comparing the binding of the specific dual tropic HIV-1 gp120 V3 loop with CCR5 and CXCR4, we observe that the HIV-1 gp120 V3 loop residues 13–21, which include the tip, share nearly identical structural and energetic properties in complex with both coreceptors. This result paves the way for the design of dual CCR5/CXCR4 targeted peptides as novel potential anti-AIDS therapeutics.

## Introduction

The first step of the Human Immunodeficiency Virus type 1 (HIV-1) cell entry comprises the interaction of the envelope glycoprotein gp120 with the host leukocyte glycoprotein receptor, CD4, and the binding to chemokine receptors CCR5 or CXCR4 [1]–[8]. As a result of the interaction of glycoprotein gp120 with CD4, the third variable region (V3) loop of gp120 is exposed [9], and subsequently, it binds to chemokine receptors CCR5 or CXCR4, infecting mostly CD4+ T-cells [1], [2]. The molecular recognition of chemokine receptors is predominantly mediated through the V3 loop fragment of HIV-1 gp120 [10]–[12]. Upon the V3 loop-coreceptor binding, a series of rearrangements in the envelope glycoproteins occur which lead to the fusion of the host and virus cell membranes [3], [4].

Following the discovery of the key role of the HIV-1 gp120 V3 loop in altered tropism [1], [13], recognizing CXCR4 or CCR5 or both (referred as “dual tropic”), several studies aimed at elucidating the key interacting residues of chemokine receptors involved in the V3 loop binding, through the mapping of the chemokine receptors and HIV-1 gp120 binding sites [10]–[12], [14]–[29]. Recently, we reported the first complete HIV-1 gp120 V3 loop : CXCR4 complex structure using molecular dynamics (MD) simulations and free energy calculations [30]. Owing to the remarkable agreement of the derived structure with previous experimental findings, the computationally derived structure elucidated the key interactions between the HIV-1 gp120 V3 loop and CXCR4 which are associated with the HIV-1 coreceptor activity [30].

The HIV-1 gp120 V3 loop is encountered in a large sequence variability and is predominantly composed of 35 residues which are connected through a disulfide bridge between residues 1 and 35 [31], [32]. Due to its highly dynamic character, the unbound V3 loop is absent in the majority of gp120 crystallographic structures; nevertheless, it was resolved in two crystallographic PDB entries [7], [8]. On the contrary, our recent study [30] revealed that, at least for the specific dual tropic V3 loop in complex with CXCR4, the V3 loop bound conformation is well defined and tight, and in addition, the loop adopts a maximized tip-base conformation, one of the key unbound V3 loop conformations identified in [31]. Similarly, Pan *et al.*, showed that understanding the unbound properties of gp120 domains is important for delineating the mechanism of conformational changes from unbound to bound structures, related to the gp120 : CD4 binding [33], [34]. The absence of an experimental structure revealing the HIV-1 gp120 V3 loop : CCR5 interaction could be associated with the high flexibility of the V3 loop leading to absence of electron density in the gp120 crystal structures, as in [35]. Several studies [8], [19], [36]–[39] attempted to computationally elucidate the molecular recognition of CCR5 by HIV-1 gp120. Nevertheless, according to our knowledge, none of the previous studies, which either considered the entire CCR5 protein [19], [36], [37], [39] or not [8], [38], resulted in a complete HIV-1 gp120 V3 loop : CCR5 structure in a high-degree of agreement with a wide spectrum of experimental findings [14]–[29] (see *Discussion*). Owing to this, the basic biological knowledge on the specific interactions between the V3 loop and one of the two chemokine receptors, CCR5, is still limited due to the absence of a complete V3 loop : CCR5 structure [40] in accordance with experiments.

Driven by our recent success in elucidating the molecular recognition of CXCR4 by a dual tropic HIV-1 gp120 V3 loop with an in-house comprehensive computational protocol [30], in this study we introduce it to delineate the molecular recognition of CCR5 by a dual tropic HIV-1 gp120 V3 loop with an identical sequence to the one used in [30]. We report here, what is to our knowledge, the first complete HIV-1 gp120 V3 loop : CCR5 structure which is in exceptional agreement with experiments. The computational protocol applied was not biased by any experimental evidence. The derived structure interprets previous experimental findings (see Table 1; marked in bold face are CCR5 residues reported in experimental findings), and thus, it sheds light on the functional role of the HIV-1 gp120 V3 loop and CCR5 residues associated with the HIV-1 coreceptor activity. This work provides insights into the blocking mechanism of HIV-1 gp120 by maraviroc, and the HIV-1 coreceptor selectivity. By comparing the binding of the specific dual tropic HIV-1 gp120 V3 loop with CCR5 and CXCR4 [30], we observe that the HIV-1 gp120 V3 loop residues 13–21, which include the tip, share nearly identical structural and energetic properties in complex with both coreceptors. This result paves the way for the design of dual CCR5/CXCR4 targeted peptides as novel potential anti-AIDS therapeutics, which would mimic the 13–21 HIV-1 gp120 V3 loop binding in complex with both coreceptors.

**Table 1:** Important intermolecular polar and non-polar interaction free energies, hydrogen bonds, salt bridges, between V3 loop and CCR5 residue pairs within the MD simulation of the complex with the lowest average binding free energy (see *Methods*).

V3 loop Residue^§^	CCR5 Residues (Polar, Non Polar Interaction Free Energies)^¶^	Salt Bridges† and Hydrogen Bonds‡
Arg3	Ile12 (−0.8, −0.6)^‡^, **Tyr15** (−3.3, −4.0)	‡Arg3 NH2 : Ile12 O, ‡Arg3 NH2 : **Tyr15** OH
Pro4	**Tyr15** (−0.1, −0.7)	
Asn5	**Asp11** (−0.5, −1.2), Ile12 (−0.3, −1.6), **Asn13** (−0.7, −0.6), **Tys14** (−1.8, −1.9), **Tyr15** (−2.1, −2.1), **Glu18** (−2.5, −0.8)	‡Asn5 ND2 : **Asp11** O, ‡Asn5 ND2 : Ile12 O,‡ Asn5 OD1 : **Tys14** N,‡ Asn5 OD1 : **Tyr15** N
Asn6	**Tys14** (−0.5, −1.5), **Glu18** (−7.3, −1.9)	‡Asn6 N : **Glu18** OE2, ‡Asn6 ND2 : **Glu18** OE*
Asn7	**Tys3** (−0.2, −1.5), **Gln4** (−0.2, −2.4), **Tys14** (−6.6, −3.7)	‡Asn7 OD1 : **Gln4** N, ‡Asn7 ND2 : **Tys14** OS4
Thr8	**Tys14** (−3.3, −3.6), **Glu18** (−0.9, −2.5), **Phe264** (−0.1, −0.6)	‡Thr8 N : **Tys14** SO4, ‡Thr8 OG1 : **Glu18** O
Arg9	**Asp2** (−13.1, −.2), **Gln4** (−1.8, −3.4), **Pro183** (−5.1, −3.4), **Tyr184** (−1.5, −5.8), **Tyr187** (1.2, −1.3), Gln188 (−1.4, −1.7), Met1 (0.6, −0.5)	†Arg9:**Asp2**, ‡Arg9 NE : **Gln4** NE2, ‡Arg9 NE/NH2 : **Gln4** OE1, ‡Arg9 NE/NH2 : **Pro183** O, ‡Arg9 NH2 : **Tyr184**
Lys10	**Tys14** (−0.6, −0.4), **Ser17** (−6.9, −0.9), **Cys20** (0.5, −1.0), Gln188 (−2.7, −1.5), **Lys191** (−4.1, −0.8), Gln261 (−2.9, −1.8), Glu262 (−2.3, −2.0), **Phe264** (−1.1, −2.3), Ser272 (−1.2 −0.4)	‡Lys10 NZ : **Ser17** O, ‡Lys10 NZ : **Ser17** OG, ‡Lys10 N : Gln188 OE1, ‡Lys10 O : **Lys191** NZ, ‡Lys10 NZ : Gln261 O, ‡Lys10 NZ : Ser272 OG
Arg11	Gln170 (−3.5, −2.5), **Glu172** (−2.7, −1.2), Ser179 (−3.0, −3.3), Ser180 (−12.8,1.7), **His181** (−0.1, −2.0), **Pro183** (−0.4, −2.2), **Tyr184** (−0.9, −2.9), **Lys191** (1.7, −2.3), **Phe182** (0.2, −0.4)	‡Arg11 NH1/NE : Gln170 OE1, ‡Arg11 NE/NH1 : Gln170 NE2, †Arg11: **Glu172**, ‡Arg11 NH1/NH2 : Ser179 OG, ‡Arg11 NH2/NH1 : Ser180 O, ‡Arg11 NH1 : **His181** ND1, ‡Arg11 NE : **Tyr184** OH
Val12	Ser179 (−2.6, −1.8), Ser180 (−0.3, −1.6), **Lys191** (−0.8, −1.6)	‡Val12 N : Ser179 OG
Ser13	Asn24 (−0.4, −0.5), Gln170 (0.1, −0.8), **Glu172** (−0.2, −0.8), Thr177 (−3.7, −1.5), **Cys178** (−2.1, −1.2), Ser179 (−4.8, −1.9)	‡Ser13 OG : **Glu172** O, ‡Ser13 N: Ser179 OG, ‡Ser13 OG : Thr177 OG1
Leu14	**Trp86** (0.0, −0.9), Tyr89 (−2.1, −2.2), Thr177 (−1.3, −2.2), **Cys178** (−2.5, −2.3), Leu104 (0.1, −0.3)	‡Leu14 O : Tyr89 OH, ‡Leu14 N : **Cys178** O
Gly15	Asn24 (0.1, −0.8), Tyr89 (0.2, −1.7), Thr177 (−0.5, −0.4)	
Pro16	**Lys26** (−0.3, −2.8), Ala30 (−0.2, −1.3), Tyr89 (0.8, −2.0), Ala90 (0.3, −2.1), **Gln280** (0.0, −1.9)	
Gly17	Leu33 (0.0, −0.6), **Tyr37** (1.5, −0.7), **Trp86** (−1.8, −2.0), Tyr89 (1.5, −1.7), Ala90 (0.3, −1.2), **Glu283** (−10.1, −0.8)	‡Gly17 N: **Trp86** O
Arg18	**Tyr37** (1.6, −1.6), Phe79 (0.3, −1.5), **Trp86** (−1.7, −3.9), **Tyr108** (−3.6, −1.9),**Phe112** (0.2, −0.3), **Trp248** (−0.9, −1.3), **Tyr251** (−3.3, −3.2), **Glu283** (−74.1, 3.0), Gly286 (−10.8,0.9), Met287 (0.8, −1.6), His289 (−15.5, 0.8)	‡Arg18 NE : **Tyr37** OH, ‡Arg18 NH1 : **Tyr108** OH, ‡Arg18 O : **Tyr251** OH, ‡Arg18 N : **Glu283** OE*, †Arg18:**Glu283**, ‡Arg18 NH2 : Gly286 O, ‡Arg18 NH1 : His289 NE2
Val19	**Tyr251** (−0.2, −1.0), Met279 (−0.3, −3.3), **Gln280** (0.1, −2.3), **Glu283** (0.1, −1.3)	
Trp20	Thr195 (0.1, −1.1), **Ile198** (0.2, −0.7), **Tyr251** (−0.2, −1.0), **Leu255** (0.0, −2.0), Asn258 (−0.5, −2.3), Thr259 (0.0, −0.8), Glu262 (0.1, −0.6), Met279 (−0.7, −2.8)	‡Trp20 NE1 : **Tyr251** OH, ‡Trp20 N : Met279 SD
Tyr21	**Lys22** (−0.8, −3.4), Ile23 (−2.9, −0.6), Asn24 (−1.4, −4.1), Val25 (−0.8, −2.4), **Asp276** (−3.0, −3.9), Gln277 (−0.6, −0.9), **Gln280** (0.0, −1.2)	‡Tyr21 OH : Ile23 O, ‡Tyr21OH : Val25 N
Thr22	**Lys22** (−5.1, −1.1), Ser272 (−0.3, −1.5), **Asp276** (−13.6, −0.3), Asn273 (−0.1, −0.4)	‡Thr22 O : **Lys22** NZ, ‡Thr22 N : **Asp276** OD*, ‡Thr22 OG1 : **Asp276** OD*
Thr23	**Cys20** (−0.9, −1.1), **Gln21** (−2.7, −2.3), **Lys22** (−2.4, −2.3) Gly173 (−0.1, −0.9)	‡Thr23 OG1 : **Lys22** N, ‡Thr23 OG1 : **Lys22** O
Gly24	**Cys20** (−0.8, −1.0), **Gln21** (−2.3, −2.4)	‡Gly24 N : **Cys20** O, ‡Gly24 O : **Gln21** NE2
Gln25	**Gln21** (−0.1, −1.5), **Glu172** (−2.7, −2.3), **Tyr184** (−3.5, −0.9)	‡Gln25 NE2 : **Glu172** OE*, ‡Gln25 OE1 : **Tyr184** OH, ‡Gln25 NE2 : **Tyr184** OH
Ile26	**Glu18** (−0.1, −1.4), **Pro19** (0.0, −1.1), **Gln21** (0.0, −1.4)	
Val27	Met1 (0.0, −2.3), **Asp2** (0.1, −0.7), **Gln4** (0.0, −0.9)	
Asp29	Met1 (−28.5, 0.9), **Asp2** (−3.6,−0.6), **Tys3** (−7.6, −6.0)	†Asp29 : Met1, ‡Asp29 OD2 : **Asp2** N, ‡Asp29 OD2 : **Tys3** N
Ile30	**Tys3** (−4.3, −2.9)	‡Ile30 N: **Tys3** OS*
Arg31	**Tys3** (1.0, −2.9), **Gln4** (−8.8, −0.7), **Val5** (−0.3, −1.7), Ser6 (0.8, −2.6), **Pro8** (−0.2, −0.3), **Tys10** (−2.1, −0.5), **Asp11** (−24.1,0.8), **Tys14** (−19.9, −1.1)	‡Arg31 NH1 : **Gln4** O, ‡Arg31 NH1 : Ser6 OG, ‡Arg31 NH2 : **Asp11** OD1, †Arg31:**Asp11**, †Arg31:**Tys14**
Lys32	**Pro8** (−0.2, −0.5), **Asp11** (−9.3, −1.5), Ile12 (−0.3, −1.9)	†Lys32 : **Asp11**
His34	Ile12 (−0.3, −3.6), **Tyr15** (0.0, −0.6)	

CCR5 residues marked in **boldface** are experimentally associated with HIV-1 coreceptor activity. The results presented correspond to analysis of 1000 snapshots of Complex 14. ^‡^Principal interacting V3 loop^§^- CCR5^¶^ residue pairs: for each ^‡^pair listed in the column, the average polar and nonpolar average interaction free energies (polar, nonpolar), are provided in parentheses next to each CCR5 residue; all energies are in kcal/mol.

† Salt bridges between V3 loop and CCR5 residue pairs.

‡ Hydrogen bonds between V3 loop and CCR5 atom pairs.

The asterisk (*) symbol used after any V3 loop/CCR5 atom in the hydrogen bonding pair denotes that any of the atoms in the charged, carboxyl or amide, side-chain group can participate in the hydrogen-bond formation.

## Methods

### Modeling, Free Energy Calculations and Molecular Dynamics (MD) Simulations:

The methodology used in the present study to derive the HIV-1 gp120 V3 loop : CCR5 complex structure consists of the following principal steps: 1) Modeling and production of flexible templates for the V3 loop and CCR5 using MD simulations; 2) Docking of selected V3 loop structures to selected CCR5 structures; 3) First round of energy minimization and binding free energy calculations of the docked complexes using the membrane GBSA approximation; 4) Second round of energy minimization and binding free energy calculations of the docked complexes using the membrane PBSA approximation; 5) MD Simulations of the docked complexes acquiring the lowest binding free energy of the previous step; and 6) Binding free energy calculations of the complex structures produced in the MD simulations to identify the complex structure with the lowest average binding free energy.

#### 1) Modeling and production of flexible templates for the V3 loop and CCR5 using MD simulations

We investigated the following dual tropic V3 loop sequence CTRPNNNTRKRVSLGPGRVWYTTGQIVGDIRKAHC, which is deposited in the Los Alamos National Laboratory database (http://www.hiv.lanl.gov). The specific V3 loop sequence of subtype B is extracted from a Chinese patient and obeys the “11/24/25” rule [41]. The V3 loop modeling, production of flexible templates, as well as the selection of the most representative structures for subsequent use in the docking procedure was previously performed and analytically explained in [30]; in summary, the replica exchange molecular dynamics simulation protocol using the FACTS [42] implicit solvent model, as in [30], [43]–[45], was applied to produce multiple templates for the V3 loop. As for CCR5, owing to (i) the high degree of homology and structural similarity between CXCR4 [46] and CCR5 [38], especially within the transmembrane (intramembrane) helical region, (ii) our success in constructing the complete CXCR4 in complex with a V3 loop, with a correct relative orientation between the N-terminal domain and transmembrane domains, and (iii) the fact that maraviroc in the CCR5 structure is an allosteric inhibitor which may induce conformational changes to CCR5 that impede gp120 binding [38], we used MEDELLER [47] to construct three preliminary V3 loop : CCR5 complexes based on [30]. The three preliminary V3 loop : CCR5 docked complexes corresponded to the three lowest binding free energy complexes identified in *Step 7* of Tamamis and Floudas [30]. A similar approach to model an (incomplete) V3 loop binding to CCR5 (with a missing N-terminal domain) was applied very recently by Tan *et al.* [38] where, despite the presence of an X-ray CCR5 structure in complex with maraviroc, a homology model was used for CCR5 [38]. In our case we exploited (i) the high homology and structural similarity between CXCR4 [46] and CCR5 [38], as well as (ii) the optimum “wide-opening” of the binding pockets of our recently published CXCR4 structures with regard to accommodating the V3 loop [30], to construct the CCR5 conformations, which would also be optimized to accommodate the V3 loop. The N-terminal domain of CCR5 was carefully modeled so as to maintain the correct and appropriate relative orientation with regard to the receptor as in [30], and in addition, it was modified, upon sequence homology to the CXCR4 N-terminus and superposition, to be folded into the exact helical-like conformation which is deposited in PDB entry 2L87 (fragment containing gp120 bound N-terminal residues 7–23 of CCR5) [48]. FREAD was applied to model the missing loops [49], and finally, I-TASSER was applied to model all the rest missing residues [50].

As shown in what follows, the derived V3 loop : CCR5 structure of our study is in exceptional agreement with experiments, which clearly validates our modeling procedure for both CCR5 and the V3 loop. Also, a comparison between (i) the computationally derived structure of CCR5 in complex with the V3 loop (in the present study), and (ii) the X-ray structure of CCR5 in complex with maraviroc (with a resolution of 2.7 Å) [38], shows that the CCR5 conformation in both complex structures is similar, with regard to the experimentally defined HIV-1 gp120 –transmembrane – binding site of CCR5 (see *Discussion*).

We employed MD simulations to produce multiple – receptor – flexible templates for the human CCR5 chemokine receptor, and in addition, to structurally refine CCR5, with particular interest on the N-terminal segment 1–20, and provide multiple possible conformations for the flexible extracellular loops [38]. As the goal was not only to refine the structure, but also to produce flexible templates which could constitute proper receptors for docking, we considered that the use of preliminary docked V3 loop : CCR5 conformations would be most beneficial for the subsequent docking procedure, as they would maintain CCR5 in suitable conformations to be recognized by the V3 loop. As in [30], for each of the three yielded complex structures we performed two independent MD simulations to produce flexible template structures for CCR5. Within the MD simulations, the system was immersed in a heterogeneous water-membrane-water environment, represented implicitly by the switching-function generalized Born (GBSW) module [51], [52]. The MD setup and parameterization [53] which was employed is identical to the one used in Tamamis and Floudas [30]. Prior to the production runs, four heating steps of total duration 400 ps were performed, and in addition, an equilibration procedure of a total duration of 1.7 ns was performed, during which the harmonic restraints were gradually removed. The production runs were performed at 300 K with a total duration of 5 ns. The difference between the two independent simulations per complex was based on the restraints imposed during the production runs: in the first simulation, no restraints were imposed on the system, whereas in the second simulation, a weak harmonic force constant of 1 kcal/mol*A^2^ was applied to the Cα atoms using the bestfit module in CHARMM [54]. The simulations were conducted with CHARMM, version c35b6 [54].

#### 2) Docking of selected V3 loop structures to selected CCR5 structures

As for the V3 loop, we used the 20 clustered V3 loop conformations which were produced from the replica exchange MD simulations of Tamamis and Floudas in [30]. As for CCR5, we merged the CCR5 structures produced from the six independent aforesaid MD simulations in single trajectory containing 1500 snapshots of CCR5. We employed the quality clustering method of WORDOM [55], to cluster independently the CCR5 structures based on their Cα coordinates; only Cα atoms with a z-coordinate value greater than 0 Å were considered in the clustering. The cluster analysis produced 15 clusters for the receptor, including the initially modeled CCR5 structure. Subsequently, the parallel linux version of Zdock v.3.0.2 [56] was employed to dock the 20 V3 loop clustered structures to the 15 CCR5 clustered structures. For each Zdock docking run, 2000 docked structures were produced with a dense rotational sampling and a masking applied on the region with protein coordinates z<0 Å, aiming at excluding the non-potential binding region from the docking calculations. Consequently, 600,000 complex structures were produced.

#### 3) First round of energy minimization and binding free energy calculations of the docked complexes using the membrane-GBSA approximation

All 600,000 complexes were subjected to 100 steps of steepest descent minimization to alleviate bad contacts and, the binding free energy was evaluated subsequently for all complexes using the GB (Generalized Born) SA approximation in a heterogeneous water-membrane-water environment, modeled by GBSW [52]. The binding free energy is evaluated via the expression, 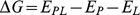, where *E*_X_ is the total (free) energy of molecule X (complex PL: CCR5:V3 loop, free protein P: CCR5, or free ligand L: V3 loop), as in [30].

#### 4) Second round of energy minimization and binding free energy calculations of the docked complexes using the membrane-PBSA approximation

Out of the 600,000 complexes, we selected the 9000 V3 loop : CCR5 complexes with the lowest GBSA binding free energy, and performed an additional round of 200 steepest descent steps energy minimization in a heterogeneous water-membrane-water environment, modeled by GBSW [52]. Subsequently, we calculated the binding free energy using the PB (Poisson Boltzmann) SA approximation, as described in [30]. At the end of this procedure, the complex structure with the lowest binding free energy −220.7 kcal/mol was identified, and additionally, we selected all the complex structures within approximately a 25 kcal/mol range of the lowest binding free energy (−220.7 kcal/mol : −195.1 kcal/mol) for subsequent investigation. As a result, the total number of complex structures selected for subsequent investigation was 19. Table S1 presents the binding free energies of the 19 different complex structures produced in step 4.

#### 5) MD Simulations of the docked complexes acquiring the lowest binding free energy

We performed 19 independent MD simulations of the complexes with the lowest PBSA binding free energies, as identified in the previous step. The MD simulations comprised a 400 ps heating procedure and an additional 700 ps equilibration procedure at which the harmonic restraints were gradually removed from CCR5 and the V3 loop. No restraints were imposed during the production run at 300 K, the duration of which was equal to 20 ns for every individual complex. The simulation methodology and force field parametrization used, was identical to step 1 at which we also performed MD simulations in an implicit membrane environment to produce flexible templates for CCR5. 1000 snapshots from each simulation, corresponding to 20 ps intervals, were used for all subsequent analyses.

#### 6) Binding free energy calculations of the complex structures produced in the MD simulations to identify the complex structure with the lowest average binding free energy

We re-evaluated the binding free energy for all the extracted simulation snapshots from all complexes using a heterogeneous water-membrane-water MM GBSA approximation, modeled by GBSW [52]. According to the calculations which are presented in Table S1, Complex 14 possessed the lowest average binding free energy. The average binding free energy of Complex 14 (−418.5 kcal/mol) is at least by a standard deviation (∼15 kcal/mol) lower than the average binding free energies of Complexes 6 (−396.7 kcal/mol) and 12 (−394.8 kcal/mol), which possess the second and third lowest binding free energy, respectively. While the MM GBSA approximation is capable of discriminating Complex 14, as the highest ranked complex for additional analysis, additional MM PBSA calculations on the highest ranked complexes according to MM GBSA (Complexes 14, 6, 12, 3, 17, 8, 18, 1, 13) showed that Complexes 14, 12, 6 and 1 fall within a 4 kcal/mol range, which is less than a standard deviation (∼10–15 kcal/mol). This can most presumably be attributed to the “step function-like” PB set up used which does not include any smoothing between the dielectric constants 2 and 80, in contrast to the more rigorous smoothing dielectric setup used in the GB module which is employed in this study. Thus, we focus our analysis in the *Results* and *Discussion* on Complex 14, as it is clearly the highest ranked according to MM GBSA, and also has remarkable agreement with experimental findings (see *Results* and *Discussion*). Nevertheless, owing to the relatively high degree of similarity between Complex 14 and the rest most highly ranked complexes, we provide analysis of the complexes and a discussion on their key differences with Complex 14 (see *Information S1*). The MD coordinates of Complex 14, extracted every 2 ns, are provided in PDB format (see *Information S2*).

### Interaction Free Energies Analysis of V3 loop : V3 loop and V3 loop : CCR5 Residue Pairs:

The interaction free energies between two residues (R and R′) in Complex 14 were computed by:
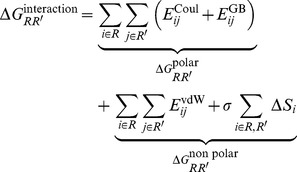
(1)The first and second group of terms on the right-hand side of Eq. (1) describe, respectively, polar and nonpolar interactions between R and R′. For the investigation of V3 loop : CCR5 intermolecular interactions, R corresponds to a V3 loop residue and R′ to a CCR5 residue. For the investigation of V3 loop intramolecular interactions, both R and R′ correspond to different V3 loop residues. To compute the GB term in Eq. (1), we set the charges of atoms outside the two – under investigation – residues R and R′ to zero. The last term contains the difference in solvent accessible surface areas of residues R and R′ in the complex and unbound states [30]. The generalized-Born energies and the atomic accessible-surface areas (ΔS_i_) entering in Eq. (1) depend on the location of R and R′ in the complex. The polar component contains a Coulombic term and a GB contribution, modeling the interaction between residue R and the solvent polarization potential induced by R′ (or vice-versa). Similarly, the non-polar component contains a van der Waals interaction between R, R′ and a surface term, expressing cavity contributions and nonpolar interactions with the surrounding solvent. The non-polar and polar solvation terms were calculated using the GBSW heterogeneous water-membrane-water representation, using the same parametrization as step 1. The sum of the two components, polar and non-polar, reflects the total direct interaction between R and R′ in the solvated complex. A similar methodology has been used for the elucidation of the molecular recognition of CXCR4 by the same dual tropic V3 loop [30] and by CXCL12 [57], the delineation of problems related to species specificity of proteins [58], the design of modified-“transgenic” proteins [59], and in problems related to drug design [60]–[62]. In our analysis, we calculated the residue pairwise interaction free energies for all 1000 snapshots in Complex 14. Subsequently, we decomposed the polar and non-polar interaction free energy contributions, and present the results of the average intra- and inter- molecular interaction free energies of the lowest binding free energy complex in two dimensional density maps in Figure S1A and Figure S2, respectively. In addition, we summed up the total intermolecular interaction free energies per V3 loop residue, as in [30], [58]–[61], so as to provide insights into the role of each interacting V3 loop residue with CCR5. Also, we provide a comparison to the sum of intermolecular interaction free energies summed up per V3 loop residue, with regard to CXCR4 binding [30] using data from the molecular recognition of CXCR4 by the same dual tropic V3 loop, by Tamamis and Floudas [30].

## Results

### Structural Properties of the Bound V3 loop:

**Figure 1:**
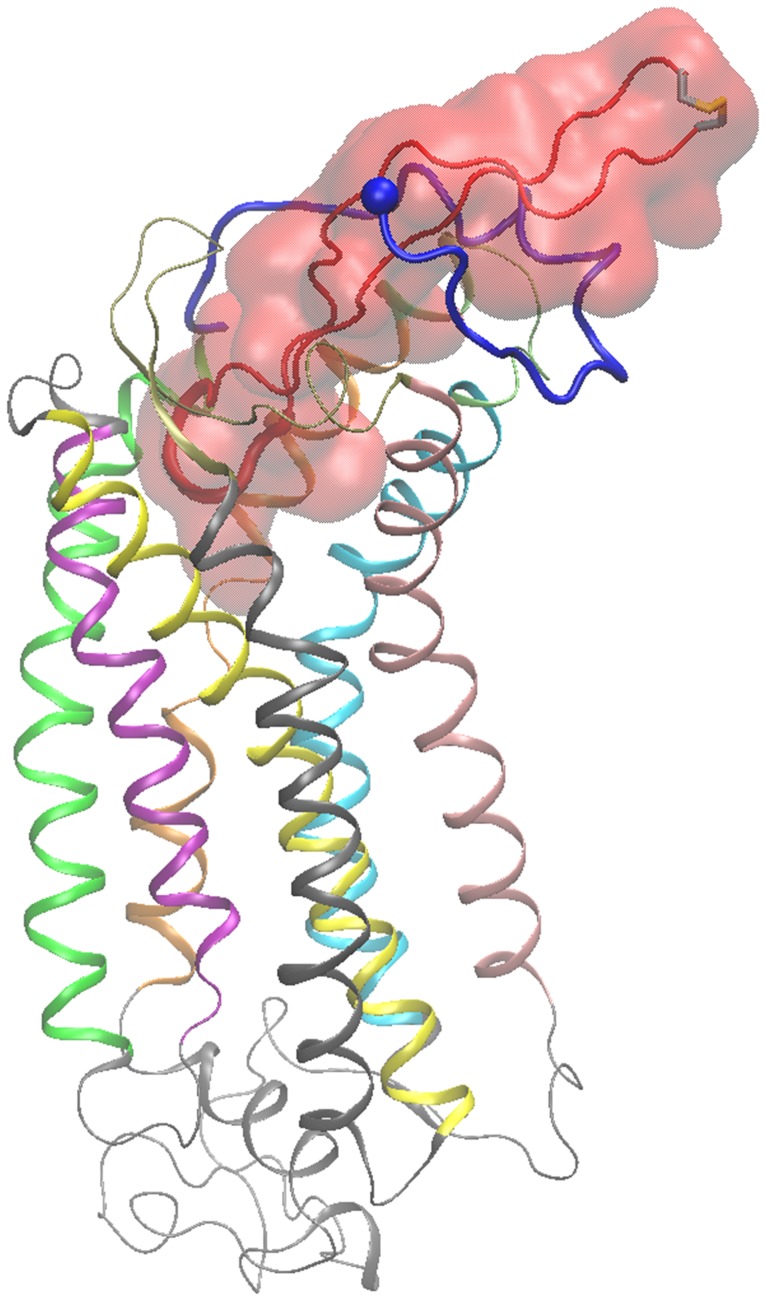
HIV-1 gp120 V3 loop : CCR5 Complex Structure; Molecular graphics image of the entire simulation system corresponding to the complex with the lowest average binding free energy. The V3 loop is shown in tube and transparent surface representation in red color, and the residue moiety 16–20 is shown in fat tube representation. The CCR5 is shown in cartoon representation, and the coloring used for different protein domains is as follows: (i) N-terminal domain is colored in blue, (ii) Transmembrane helix 1 (TH1) is colored in green; (iii) Intracellular loop 1 (ICL1) is colored in light gray; (iv) TH2 is colored in purple, (v) Extracellular loop 1 (ECL1) is colored in light gray; (vi) TH3 is colored in yellow; (vii) ICL2 is colored in light gray; (viii) TH4 is colored in gray; (ix) ECL2 is colored in ochre; (x) TH5 is colored in pink; (xi) ICL3 is colored in light gray; (xii) TH6 is colored in cyan; (xiii) ECL3 is colored in lime; (xiv) TH7 is colored in orange; (xv) C-terminal domain is colored in light gray. The N-terminal Cα atom of CCR5 is shown in a small van der Waals sphere and the V3 loop disulfide bridge is shown in fat transparent licorice representation. The definition of CCR5 and V3 loop domains is presented in *Information S4*.

The structural properties of the V3 loop bound to CCR5 are similar to the properties of the V3 loop bound to CXCR4 [30]. V3 loop residues 8–26 are buried within CCR5, whereas residues 1–7 and 27–35 mainly lie upon the N-terminal end of CCR5 (all V3 loop residues are renumbered, starting from 1 and ending at 35). The V3 loop conformation is twisted, as shown in Figure 1 and, is maintained in a β-hairpin conformation throughout the simulation. Specifically, antiparallel β-sheets among the following residue moieties, 3–11:23–34 and 14:20, are observed in the trajectory, and also, two consecutive β-turns are observed within the core of the tip comprising residues 16:20 which is the mostly buried region of the V3 loop within the CCR5 binding pocket, as shown in Figure 1, similarly to the V3 loop : CXCR4 complex structure [30]. The β-sheets provide a compact-thin shape and a stable conformation of the V3 loop within the simulation. The V3 loop residues lying outside the chemokine receptor experience a slightly higher flexibility; the average backbone RMSD without alignment with respect to the starting conformation is 2.0±0.1 Å and 1.4±0.1 Å, for the entire V3 loop and the embedded region (8:26), respectively (see Table S2). While the unbound V3 loops experience high flexibility [30], [31], [63], [64], the bound V3 loop conformations, at least for the specific dual tropic V3 loop both in complex with CCR5 and CXCR4 [30], are well defined, tight, and are interestingly mutually similar within residue moiety 8–26, and nearly identical within the residue moiety 13–21: the RMSD of the lowest binding free energy complex (V3 loop : CCR5), with regard to the last 20-ns structure of Complex 1 in (V3 loop : CXCR4) [30] is, upon superposition, 2.4±0.1 Å, 1.2±0.1 Å and 1.0±0.1 Å, for the entire V3 loop, residue moiety 8–26, and residue moiety 13–21, respectively. As in [30], both (i) the cooperativity of both intramolecular interactions in the bound structure, shown in Figure S1A, and (ii) the intermolecular interactions, which are analyzed below, contribute to the tight binding of the V3 loop. The increased stability of the 8–26 bound V3 loop conformation, which is observed both in complex with CCR5 and CXCR4 [30], could also be attributed to protein-solvent interactions, and more specifically, be associated with dewetting [65]. We calculated the average percentage of buried surface area of each V3 loop residue in complex with CCR5 or CXCR4 [30], and normalized it by the total surface accessible area of each corresponding residue in its unbound state. This analysis is presented in Figure S1B, and shows that V3 loop residues 8–23 in both complexes are nearly entirely buried owing to contacts caused by the binding to CCR5 and CXCR4 [30]. In addition, V3 loop residues 24–26 share a somewhat similar burial behavior in complex with the two coreceptors. While the almost entire burial of V3 loop residues 12–21 is not surprising, as these residues interact predominantly with the transmembrane residues of coreceptors, V3 loop residues 8–11 and 22–23, which are not located within the transmembrane region of the coreceptors, are interestingly almost entirely buried too. All in all, the complete burial of V3 loop residues 8–23 is expected to contribute to the dewetting of these residues, a mechanism which can, additionally contribute to the stability [65] of the specific V3 loop domain in complex with both CCR5 and CXCR4 [30].

### Structural Properties of the bound CCR5:

Within the simulation, the CCR5 conformation is very well retained with regard to the starting conformation *a posterior* to equilibration. The average backbone RMSD of the transmembrane helical residues is equal to 1.2±0.2 Å, and the average RMSD of the entire backbone is 2.2±0.2 Å. The larger value of the latter is attributed to the higher flexibility of the non-transmembrane domains. According to DSSP definitions [66], in approximately 90% of the simulation snapshots, CCR5 residues within 7–13 domain are predominantly folded into 3_10_, and to a smaller extent α-helices, in agreement with Schnur *et al.* [48]; a helical conformation for the N-terminal segment of CCR5 has also previously been reported in [8].

### Investigation of the Complete V3 loop : CCR5 Complex Structure:

**Figure 2:**
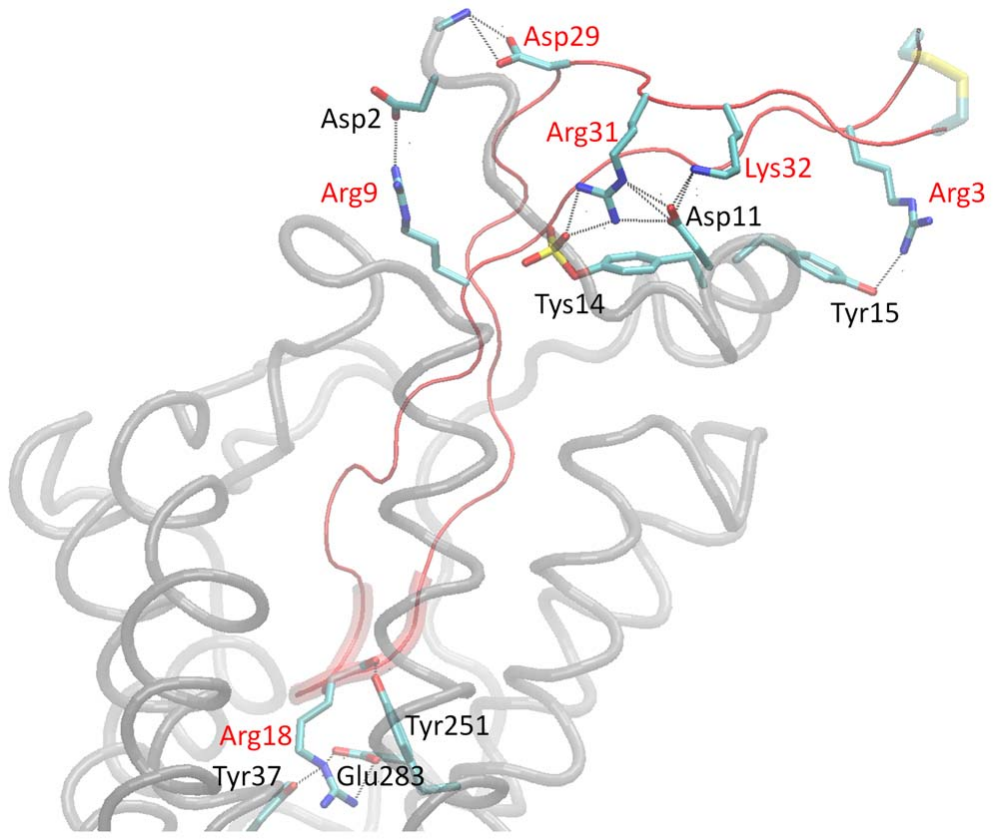
Important Intermolecular Polar Interactions; Molecular graphics image of important polar interactions corresponding to the complex with the lowest average binding free energy. The figure shows the salt bridges and specific important hydrogen bonds. The V3 loop is shown in tube and in red color, and the residue moiety 16–20 is shown in fat tube representation. The CCR5 is shown in light gray transparent tube representation. The salt bridge and hydrogen bonds are denoted in dashed lines and the participating V3 loop and CCR5 residue moieties are shown in licorice; V3 loop and CCR5 residues are annotated in red and black, color respectively. Hydrogen atoms are omitted for clarity, and the V3 loop disulfide bridge is shown in fat transparent licorice representation.

We present a detailed overview of the structural and physicochemical properties of the derived complex structure. The analysis is based on the assessment of the intermolecular residue pair-wise interaction free energies, which is shown in Figure S2, as well as hydrogen bond occupancies which are shown in Table S3. Figure 2 presents important salt bridges and hydrogen bonds encountered in the trajectory, using VMD [67]. Table 1 summarizes the interactions between V3 loop : CCR5 residues, and features in bold face the CCR5 residues which, according to experimental findings, are involved in the HIV-1 coreceptor activity.

#### Interactions of V3 loop residues 1:8 with CCR5

V3 loop residues 3–8 predominantly interact with the N-terminal segment 1:20 of CCR5. The charged amide of V3 loop Arg3 forms two hydrogen bonds with the hydroxyl group of CCR5 Tyr15 (see Figure 2 and Video S1) and the backbone carbonyl of CCR5 Ile12; the former interaction also leads to a cation-π interaction between the two residues. Pro4 of the V3 loop is engaged in low intensity non-polar contacts with the side chain of CCR5 Tyr15. V3 loop Asn5 ND2 forms hydrogen bonds with the backbone carbonyl groups of CCR5 Asp11 and Ile12, and Asn5 OD1 forms hydrogen bonds with the backbone amide of CCR5 Tyr14 and Tyr15. Asn5 of the V3 loop is polarly attracted to the charged carboxyl of CCR5 residue Glu18, and its side chain is in the vicinity of the backbone of CCR5 Asn13. The backbone of V3 loop Asn6 is proximal to CCR5 Tys14, and both the Asn6 backbone amide and Asn6 ND2 form two high occupancy hydrogen bonds with the charged carboxyl group of CCR5 residue Glu18. The V3 loop Asn7 OD1/ND2 polar atoms are hydrogen bonded to Gln4 N and Tys14 OS4, respectively; in addition, the side chain of V3 loop Asn7 forms non polar contacts with both the backbone and side chain of CCR5 Tys3. Residue Thr8 of the V3 loop is packed among Tys14 and Glu18, and as a result, its backbone amide is hydrogen bonded to CCR5 Tyr14 OS4, and on the opposite side, the Thr8 side chain hydroxyl group is hydrogen bonded to CCR5 Glu18 O. Also, the side chain Thr8 methyl group is in the vicinity of CCR5 residue Phe264.

#### Interactions of V3 loop residues 9:15 with CCR5

Residues 9–15 of the V3 loop are more buried into CCR5, compared to residues 1–8, and thus, apart from interacting with the CCR5 N-terminal domain, they additionally interact with CCR5 residues in the extracellular loop 2 (ECL2), and transmembrane helices 2 and 5 (TH2 and TH5). Arg9 of the V3 loop forms a highly interacting salt bridge with CCR5 residue Asp2 (see Figure 2 and Video S1), and its charged amide is also hydrogen bonded to CCR5 Pro183 O, and to a smaller extent to Gln4 NE2, Gln4 OE1 and Tyr184 O. The position of V3 loop residue Arg9 is additionally stabilized by (i) a hydrophobic interaction with CCR5 residue Gln188 through the non-polar side chain moieties of both residues, (ii) a weak interaction between its charged amide and CCR5 Met1 SD, and (iii) a low interacting cation-π interaction between its charged amide and the aromatic ring of CCR5 Tyr187. V3 loop residue Lys10 also forms an abundance of hydrogen bond interactions with several CCR5 residues: its backbone amide is hydrogen bonded to CCR5 Gln188 OE1, and its backbone carbonyl is hydrogen bonded to CCR5 Lys191 NZ, throughout the simulation; in addition, its charged amide is hydrogen bonded to CCR5 Ser17 O, Ser17 OG, Gln261 O and less frequently to Ser272 OG. Furthermore, V3 loop residue Lys10 is attracted to CCR5 residues Glu262, Tys14 and Phe264, and its charged group is proximal to CCR5 Cys20 SG. The charged amide of V3 loop residue Arg11 participates in a series of hydrogen bonds with the following ECL2 CCR5 residues, summarized in decreasing order of occupancy: Ser180 O, Ser179 OG, Tyr184 OH, Gln170 OE1, Ser179 OG, Gln170 NE2, Glu172 OE1, His181 ND1. Despite the oppositely charged polar repulsive interaction between V3 loop Arg11 and CCR5 Lys191, the non-polar side chain moieties of the two residues contact each other; in addition, the positively charged group of Arg11 forms non-polar contacts with the side chain of CCR5 Pro183, and is in the vicinity of CCR5 residue Phe172. V3 loop residue Val12 is involved in interactions with primarily the non-polar moieties of ECL2 CCR5 residues Ser179, Ser180, Lys191; as a consequence of the first interaction, a high occupancy hydrogen bond occurs between the backbone amide of Val12 and the hydroxyl group of Ser179. Residue Ser13 of the V3 loop is involved in polar interactions with ECL2 CCR5 residues Ser179, Thr177, Cys178 and Glu172, which include (i) two high occupancy hydrogen bonds between Ser13 N : Ser179 OG, Ser13 OG : Thr177 OG1, (ii) a low occupancy hydrogen bond between Ser13 OG : Glu172 O, and (iii) a polar attraction between the backbone amide of Ser13 and the backbone carbonyl of CCR5 Cys178. Also, the hydroxyl group of Ser13 is surrounded by the polar atoms of CCR5 Asn24 ND2 and Gln170 NE2. The backbone amide of V3 loop Leu14 forms a stable hydrogen bond with the backbone carbonyl of CCR5 Cys178, the backbone carbonyl of Leu14 is in the vicinity of the hydroxyl group of CCR5 Tyr89 which results in a low occupancy hydrogen bond, and the hydrophobic side chain of Leu14 forms non-polar contacts with the side chain moieties of CCR5 Trp86, Thr177, and to a smaller extent of Tyr104. Residue Gly15 of the V3 loop is embedded in a pocket comprising CCR5 residues Asn24, Tyr89 and Thr177.

#### Interactions of V3 loop residues 16:22 with CCR5

The 16–20 V3 loop residue moiety comprises the core of the tip, and is predominantly the most buried region of the V3 loop within CCR5, as it is also the case in complex with CXCR4 [30]. V3 loop residue Pro16 is primarily participating in hydrophobic contacts with the non-polar side chain moieties of CCR5 residues Lys26, Ala30, Tyr89, Ala90, Glu280. Gly17 of the V3 loop is surrounded by the non-polar moieties of CCR5 residues Leu33, Tyr37, Trp86, Tyr89, Ala90, and Glu283, and is infrequently hydrogen bonded to the backbone carbonyl of CCR5 residue Trp86; in addition, its backbone amide is strongly attracted to the negatively charged side chain amide of CCR5 residue Glu283. Arg18 of the V3 loop is the most buried residue within the CCR5 transmembrane domain, and also the most highly interacting V3 loop residue with CCR5, similarly to the V3 loop : CXCR4 complex structure [30]. Both the charged side chain and backbone amide groups of Arg18 interact with the oppositely charged carboxyl group of CCR5 residue Glu283 (see Figure 2 and Video S1), resulting in a highly interacting salt bridge, and a hydrogen bond interaction, respectively. The charged amide group of Arg18 also participates in four additional hydrogen bonds with CCR5 atoms Tyr37 OH (see Figure 2 and Video S1), Tyr108 OH, Gly286 O and His289 NE2. Furthermore, the backbone carbonyl of V3 loop Arg18 is consistently hydrogen bonded to the side chain hydroxyl group of CCR5 residue Tyr251 (see Figure 2 and Video S1). Moreover, the side chain moiety of Arg18 (i) is embedded in a pocket comprised of the side chains of CCR5 residues Tyr37, Phe79, Trp86, Tyr108, Tyr112 and the backbone of Met287, and (ii) forms a cation-π interaction with CCR5 residue Trp248. The hydrophobic side chain of V3 loop residue Val19 is in the vicinity of the side chains of CCR5 residues Met279, Tyr251, Gln280, Gln283. Trp20 of the V3 loop is embedded in a binding pocket comprising CCR5 residues Met279, Asn258, Leu255, Thr195, Tyr251, Thr259, Glu262 and Ile198, listed in descending order of non-polar interaction free energy magnitude. The side chain orientation of Trp20 is stabilized by two hydrogen bonds between Trp20 N: Met279 SD and Trp20 NE1 : Tyr251 OH. Residue Tyr21 of the V3 loop is buried in a pocket comprised of primarily the non-polar moieties of CCR5 residues Asp276, Asn24, Lys22, Ile23, Val25, Gln277 and Gln280, listed in descending order of interaction free energy magnitude; its proximity to residues Ile23 and Val25 is facilitated by the presence of two hydrogen bond interactions between the hydroxyl group of Tyr21 with the (i) backbone carbonyl of Ile23, as well as (ii) the backbone amide of Val25. V3 loop residue Thr22 forms non-polar contacts with the side chain moieties of CCR5 residues Cys20, Ser272 and Asn273, and is also involved in hydrogen bonds between V3 loop and CCR5 atoms Thr22 O : Lys22 NZ, Asp276 OD1/2 and Thr22 OG1 : Asp276 OD1/2; these hydrogen bonds are facilitated by an intramolecular salt bridge between CCR5 residues Lys22 and Asp276.

#### Interactions of V3 loop residues 23:35 with CCR5

V3 loop residue Thr23 intercalates among - primarily - the non-polar moieties of CCR5 residues Cys20, Gln21, Lys22 and Gly173, and its hydroxyl side chain group participates in hydrogen bond interactions with the backbone moiety of CCR5 residue Lys22. V3 loop residue Gly24 is proximal to CCR5 residues Cys20 and Gln21, owing to hydrogen bonds between Gly24 N : Cys20 O and Gly24 O : Gln21 NE2. The polar side chain of V3 loop Gln25 participates in two intermolecular concurrent hydrogen bonds, Gln25 NE2 : Glu172 OE1/2 and Gln25 OE1/NE2, and is also in the vicinity of CCR5 residue Gln170; also, the backbone of V3 loop Gln25 is proximal to the side chain of CCR5 residue Gln21. Residues in the 26–35 moiety of the V3 loop lie on the upper part of CCR5, and thus, they interact solely with the N-terminal segment of CCR5. V3 loop residues Ile26 and Val27 form hydrophobic contacts with primarily the non-polar side chain moieties of CCR5 residues, Glu18, Pro19, Gln21 and Met1, Asp2, Gln4, respectively. While the side chain of CCR5 Met1 is partly solvent exposed and partly interacting with V3 loop residue Val 27, the charged N-terminal end of Met1 forms a salt bridge with V3 loop residue Asp29 (see Figure 2 and Video S1); this interaction could be most important for the stability of the N-terminal domain CCR5, and consequently, for the tight binding of the HIV-1 gp120 V3 loop into CCR5. The attraction of Asp29 to the first N-terminal domain residues of CCR5 is also facilitated by the hydrogen bond interactions between Asp29 OD2 and CCR5 backbone amide groups of Asp2 and Tys3 throughout the simulation; due to the latter hydrogen bond, the non-polar moiety of Asp29 forms highly interacting contacts with the aromatic group of CCR5 Tys3. Furthermore, the backbone of Ile30 is attracted to the negatively charged group of CCR5 residue Tys3 through hydrogen bond interactions between the backbone amide of Ile30 and Tys3 OS(2–4). Residue Arg31 of the V3 loop is the second most highly interacting of the V3 loop as it participates in an abundance of intermolecular salt bridges, hydrogen bonds and non-polar interactions with N-terminal CCR5 residues Tys3, Gln4, Val5, Ser6, Tys10, Asp11 and Tys14. Specifically, Arg31 forms two simultaneous and highly interacting salt bridges with CCR5 residues Asp11 and Tys14 (see Figure 2 and Video S1), and its charged amide group forms a high occupancy hydrogen bond with the backbone carbonyl of CCR5 Gln4. In addition, the charged amide group of V3 loop Arg31 is in the vicinity of the oppositely charged group of Tys10 and the hydroxyl group of Ser6, and the non-polar side chain moiety of Arg31 forms contacts with the hydrophobic groups of CCR5 residues Tys3, Val5 and Pro8. Residue Lys32 of the V3 loop is in the majority of the simulation frames participating in a salt bridge with CCR5 residue Asp11 (see Figure 2 and Video S1); the orientation of its side chain is additionally stabilized by a contact between its non-polar moiety and CCR5 residues Pro8 and Val12. V3 loop His34 is frequently attracted to CCR5 residue Ile12 and less frequently to CCR5 residue Tyr15. As in [30], the V3 loop Cys1-Cys35 disulfide bridge points toward the aqueous extracellular environment throughout the simulation, as would be the case if it was covalently bonded to the entire gp120 protein.

### Comparison of V3 Loop Interacting residues in Complex with CCR5 versus CXCR4: Insights from the Specific Dual Tropic V3 Loop:

**Figure 3:**
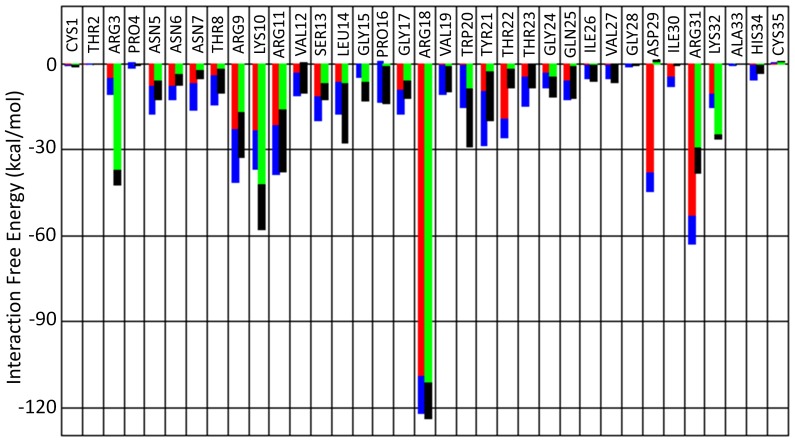
Intermolecular Interaction Free Energies of V3 loop Residues in Complex with CCR5/CXCR4; Average intermolecular interaction free energies (y-axis) of V3 loop residues (x-axis). The intermolecular interaction free energies for every V3 loop residue are summed up for all interacting residues of CCR5 (first bar per residue) and CXCR4 (second bar per residue). The polar contribution is denoted in red and green color, for CCR5 and CXCR4, respectively, and the non-polar contribution is denoted in blue and black color, for CCR5 and CXCR4, respectively. The total interaction free energy of each V3 loop residue corresponds to the sum of polar and non-polar contributions.

To obtain insights into the role of each specific V3 loop residue, and delineate the similarities and differences with regard to binding to CCR5 versus CXCR4, for the specific dual tropic V3 loop, we summed up the polar and non-polar intermolecular interaction free energies per V3 loop residue in complex with CCR5 and CXCR4 [30]. Figure 3 presents the decomposition of intermolecular interaction free energies, with regard to CCR5 (first bar per V3 loop residue) and CXCR4 [30] (second bar per V3 loop residue) binding. According to the results, the most highly interacting V3 loop residue in complex with both coreceptors is Arg18. Its interaction free energy is comparable in both complexes, and the high interaction free energy value is attributed to the strongly interacting and conserved salt bridges formed with CCR5 residue Glu283, and CXCR4 residues Asp171 and Glu288 [30]. In addition, V3 loop residues Asp29, Arg31, Thr22, Asn7 are significantly more interacting in complex with CCR5 compared to CXCR4. On the contrary, V3 loop residues Arg3, Lys10, Trp20 and Lys32 are significantly more interacting in complex with CXCR4 compared to CCR5. These differences, which depend on the sequence of the specific dual tropic V3 loop, are a consequence of variabilities associated with mainly polar interactions (e.g., salt bridges and hydrogen bonds). It is worth noting that, at least for the specific dual tropic V3 loop, seven out of eight residues with the highest degree of dissimilarity with regard to their interactions with the two coreceptors (|ΔG^CCR5-CXCR4^|>11 kcal/mol), are outside the key penetrating region 13–21 of the V3 loop, which includes the core of the V3 loop tip moiety 16:20. In general, within the 11–19 residue moiety, the V3 loop residues share similar interaction free energies with regard to the binding to both coreceptors. Residues Pro16, Arg11, Val19 and Arg18 share similar interaction strength, while Leu14 and Gly15 are slightly more interacting in the CXCR4 complex, and Ser13 is more interacting in the CCR5 complex. Outside of the 13–21 V3 loop region, residues Val12, Gln25, Ile26 and Val27 share similar total interaction potencies in complex with both coreceptors. In addition, the intermolecular polar interaction free energy of residues Gln25, Asn6, Thr23, Ile30, Thr8 and Asn5 is more favorable in the CCR5 complex, indicating that these residues are less favored to form intermolecular hydrogen bonds in complex with CXCR4.

## Discussion

### Exceptional Agreement with Experiments – Systematic Methodology Applied

Since 1996, a series of experimental studies aimed at exploring the key CCR5 and HIV-1 gp120 residues related to the HIV-1 infection due to the interaction between CCR5 and HIV-1 gp120 [14]–[29]. The V3 loop is the key determinant of HIV-1 gp120 in its interaction with the entire CCR5 [10]–[12]. The bridging sheet region of gp120, which is a four-stranded antiparallel β-sheet that includes the V1/V2 stem and two strands derived from the C4 region, has a less significant role in the HIV-1 gp120 : CCR5 interaction [10], [11]. Despite the variances with respect to the sequence of the HIV-1 gp120 V3 loop used in each of the studies, the experimental results provide unambiguous evidence on the key CCR5 residues which are important or involved in the HIV-1 coreceptor activity. We evaluated our findings in the context of a wide spectrum of available experimental data, associated with the key CCR5 residues with regard to HIV-1 binding. The overall comparison between our findings and experimental data shows that this is, according to our knowledge, the first complete HIV-1 gp120 V3 loop : CCR5 structure which exhibits exceptional agreement with experimental findings. Therefore, the results of this study are capable of shedding light into the key HIV-1 gp120 V3 loop and CCR5 residues involved in the binding and HIV-1 coreceptor activity.

#### Role of the N-terminal domain of CCR5

The N-terminal domain of CCR5 is required for HIV-1 activity, as its deletion abrogates its activity [22]. In line with this, we show that the N-terminal domain of CCR5 participates in important interactions with the HIV-1 gp120 V3 loop. Residue Asp2 of CCR5 is deemed important for HIV-1 coreceptor activity [14], [16], [25], [29], and in our computationally derived complex structure, Asp2 forms a highly interacting salt bridge with residue Arg9 of the V3 loop, and interacts with Val12 of the V3 loop. Residue Tys3 of CCR5 is also deemed important for coreceptor activity [14], [20], [25], [29] and, according to our results, the side chain of Tys3 forms polar and non-polar interactions with Ile30, and Asn7, Asp29, Arg31, respectively. In accordance with experiments [29], we show that Gln4 and Val5 of CCR5, are also involved in the HIV-1 gp120 binding. Pro8 of CCR5 is involved in the HIV-1 gp120 binding as an alanine mutation influences the binding [15]; apart from the fact that the alanine mutation can significantly modify the φ/ψ dihedral angles and the orientation of the 1–7 CCR5 N-terminal domain, Pro8 within the simulation forms non-polar contacts with V3 loop residues Arg31 and Lys32. Several experimental studies indicated that CCR5 residue Tys10 is involved in the HIV-1 coreceptor activity [14], [15], [19]–[21], [25], and more specifically, a study showed that an alanine mutation significantly reduces the HIV-1 activity for dual tropic viruses [20]. In our simulation, the charged group of Tys10 side chain is, after the first 2 ns, consistently within approximately 6.5 Å proximal to any of the charged amide atoms of V3 loop residue Arg31; this polar attraction could be significant with regard to providing an appropriate orientation for the V3 loop during the binding. Also, the Tys10 side chain charged group is throughout the simulation hydrogen bonded to the backbone amide groups of CCR5 residues Ser7 and Phe187, and these interactions could contribute significantly to the stabilization of the N-terminal domain, and also the relative orientation between the N-terminal domain and ECL2, for the HIV-1 binding to occur. Residue Asp11 of CCR5 is considered by a large number of studies critical for HIV-1 activity [14]–[16], [18], [25], [26], as alanine mutations significantly decrease the activity for HIV-1 binding; specifically, in Doranz *et al.* [26], the authors perform alanine mutations at charged residues of positions 2, 11, 18, 22, 26, 31 of the N-terminal domain, and show that the Asp11Ala mutation is by far the most critical residue for HIV-1 binding. Our results conform to this as Asp11 is involved in two highly interacting salt bridges with V3 loop residues Arg31 and Lys32. Furthermore, the weak interactions formed between Asn13 of CCR5 and the V3 loop residue Asn5 within the simulation comply with the minor role of the former residue in the binding [14]–[16], [25]; an alanine mutation at position 13 reduces the efficiency of HIV-1 entry to a relatively small extent (∼25–30%) [15]. Residue Tys14 of CCR5 is one of the most important coreceptor residues for HIV-1 activity [14], [15], [20], [21], [27], as both an alanine or a phenylalanine mutation cause a significant reduction to the HIV-1 coreceptor function [20], [27]. This finding (i) shows that both the aromatic group and the negatively charged group of Tys14 play a key role in the HIV-1 gp120 binding, and (ii) complies with additional experiments showing that an asparagine substitution also abrogates the HIV-1 coreceptor activity [28]. Our work provides a compelling evidence for this, as Tys14 forms a highly interacting salt bridge with V3 loop residue Arg31, its charged side chain group is additionally hydrogen bonded to V3 loop residue Thr8, while its aromatic group forms non-polar contacts with V3 loop residues Asn6, Lys10. Furthermore, the neighboring CCR5 residue Tyr15 is considered important for HIV-1 coreceptor activity [14], [20], [21]; while an alanine mutation causes a significant reduction to the HIV-1 coreceptor function, a phenylalanine mutation retains sufficiently the coreceptor function [20]. This finding shows that aromaticity at position 15 is critical, but the side chain hydroxyl group possesses an additional role to the binding, too. In line with this, Tyr15 OH within the simulation forms a hydrogen bond with V3 loop Arg3, and the aromatic group of Tyr15 participates in hydrophobic contacts with V3 loop residues Arg3, Pro4 and His34. Moreover, the cooperativity of the backbone carboxyl and side chain hydroxyl groups of CCR5 Ser17 in the formation of hydrogen bonds with the charged amide of V3 loop residue Lys10 can justify the involvement of CCR5 residue Ser17 in the HIV-1 binding [15], [20].

Farzan *et al.* [21] examined two different HIV-1 viruses and showed that for one virus an alanine mutation to Glu18 caused an approximately 50% reduction of the activity, while for the other virus the same mutation markedly impaired the HIV-1 function. Our simulations show that the charged carboxyl group Glu18 is predominantly hydrogen bonded to V3 loop Asn6 N/ND2. A recent study investigating the Pro19Ala mutation in CCR5 showed that Pro19 is involved in the HIV-1 binding, and accordingly, we show that Pro19 is hydrophobically attracted to V3 loop residue Ile26. Similarly to Glu18, an alanine mutation at CCR5 residue Cys20 can impair the HIV-1 coreceptor activity, or it may cause reduction to some extent, for different HIV-1 viruses 15,20. Apart from the key role of the Cys20–Cys269 disulfide bridge with regard to the structural stabilization of the receptor, our work depicts that Cys20 participates in key polar and non-polar interactions with V3 loop residues Lys10, Thr22, Thr23 and Gly24. A study showed that CCR5 residues Gln21 and Lys22 are important for activity [21], as alanine mutations at these positions correlate with a significant reduction in HIV-1 coreceptor activity. Our study provides evidence for this, as the side chain atoms of residues Gln21 and Lys22 are involved in highly interacting hydrogen bonds with V3 loop residues Gly24 and Thr22, respectively, and are also involved in contacts with V3 loop residues Thr23, Gln25, Ile26 and, Tyr21, Thr23, respectively. Our study shows that residue Lys26 of CCR5 forms a non-polar contact with V3 loop residue Pro16; this is in accordance with two experimental studies depicting that alanine [16] or glycine mutations [19] at CCR5 position 26 affect the HIV-1 binding to a small extent.

#### Role of the Transmembrane Helices 1, 2, 3, 4 of CCR5

The Arg31Gly mutation on CCR5 has no effect in the coreceptor function [19], and in line with this, our results show that Arg31 is not in the V3 loop binding site. Alanine mutations at Tyr37 of CCR5 cause a reduction in the HIV-1 gp120 binding; in our computationally derived structure, Tyr37 forms polar and non-polar interactions with V3 loop residue Arg18. An alanine mutation at position Trp86 of CCR5 decreases to a large extent the HIV-1 coreceptor activity [17]; according to our complex structure, Trp86 of CCR5 participates in significant non-polar interactions with V3 loop residues Leu14, Gly17 and Arg18. In the same study [17], the authors investigated the effect of an alanine mutation on Trp94 (of extracellular loop 1) and showed that it is also important for HIV-1 coreceptor activity; despite the fact that in our complex structure, Trp94 is not in the binding site, it forms highly conserved π-π interactions with CCR5 residue Trp86 (of TH1) which acquires a key role in the binding site as it is involved in polar and non-polar interactions with V3 loop residues Leu14, Gly15, Pro16 and Gly17. The aromatic interactions between Trp94 : Trp86 are also present in the X-ray structure [38]. Alanine mutations at CCR5 aromatic residues Tyr108, Phe109 and Phe112 decrease the HIV-1 binding activity, with the most important decrease occurring at position Tyr108 [17]. According to our derived complex V3 loop: CCR5 structure, as well as the X-ray CCR5 structure [38], these residues form intramolecular interactions through their aromatic groups, and as a result this facilitates (i) the aromatic groups of Tyr108 and Phe112 to be proximal to the non-polar moiety of V3 loop residue Arg18, as well as (ii) the hydroxyl group of Tyr108 to be hydrogen bonded with V3 loop residue Arg18.

#### Role of the Extracellular Loop 2 and Transmembrane Helices 5 and 6 of CCR5

A study showed that simultaneous alanine substitutions on CCR5 residues Lys171 and Glu172 influence of HIV-1 activity [18]. A recent study investigated the Lys171Ala and Glu172Ala mutants independently, and showed that residue Glu172 is the one involved in the HIV-1 gp120 binding, but not to a significant extent [15]. In approximately the first 2 ns of the simulation, Glu172 forms a salt bridge with V3 loop residue Arg11, and throughout the simulation it forms a hydrogen bond with V3 loop residue Gln25; thus, it is possible that during the binding process, this salt bridge could – at first – occur for the V3 loop to be accommodated in the binding site, and subsequently, this interaction can be replaced by new polar interactions, which correlate with an overall stronger binding of the V3 loop to CCR5. Experimental studies showed that an alanine substitution of CCR5 residue Cys178 reduces the HIV-1 gp120 coreceptor activity significantly [15], [17], [18], [23]. Apart from the Cys178 key role in the relative orientation of TH3 and ECL2 domains through the disulfide bridge Cys100-Cys178, Cys178 is part of the binding site in our complex structure and interacts with V3 loop residues Ser13 and Leu14. Alanine mutations at CCR5 His181, depending on the virus type, can cause a decrease in the HIV-1 coreceptor activity [15], [16], [21]; within our simulations, the side chain of His181 forms a hydrogen bond with the charged amide of V3 loop residue Arg18. Moreover, alanine mutations at CCR5 residues Phe182 and Pro183 showed that they are involved in the HIV-1 gp120 binding [15], [23]; according to our study, this can be attributed to their interactions with V3 loop residues Arg9 and Arg11. Furthermore, experiments showed that an alanine mutation at CCR5 residue Tyr184 influences the HIV-1 gp120 binding, and in line with this, we show that its side chain hydroxyl group is involved in hydrogen bond interactions with V3 loop residues Arg11 and Gln25. Experimental studies showed that alanine mutations on aromatic CCR5 residues Tyr187 [15], [23], Phe189 (of TH5) [17], [23], and Tyr190 (of TH5) [17], [23], Phe193 (of TH5) [15], [23] may in general, and depending on the virus type, affect or not HIV-1 binding. According to our computationally derived structure and the X-ray CCR5 structure [38], with the exception of Tyr187, these residues point toward the exterior of the receptor and do not belong to the experimentally defined V3 loop binding site; nevertheless, they participate in intramolecular π-π interactions which stabilize their relative orientation and preserve the integrity of the structure. Our results depict that the aromatic group of Tyr187 points toward the binding site, and forms a cation-π interaction with V3 loop residue Arg9.

CCR5 residues Lys191 [18] and Ile198 [17], [18] are involved in the binding, as the HIV-1 coreceptor activity is approximately halved due to alanine mutations at these positions. We provide evidence for the role of both residues: (i) the charged amide of Lys191 is strongly hydrogen bonded to V3 loop residue Lys10, and the non-polar side chain moiety of Lys191 forms non-polar contacts with V3 loop residue Arg11 and Val12; (ii) the hydrophobic side chain of Ile198 is strongly attracted to the aromatic group of V3 loop residue Trp20 [17]. A recent study showed that CCR5 residues Trp248, Tyr251 and Leu255 are important for HIV-1 activity. Specifically, an alanine mutation at Leu255 reduces HIV-1 activity by approximately 70%, while alanine mutations at positions 248 and 251 abrogate HIV-1 coreceptor activity. Also, even a phenylalanine substitution at position 251 reduces the activity by approximately 60%, showing that both the aromatic and the side chain hydroxyl group of Tyr251 are significant for the HIV-1 binding. Our results provide compelling evidence for the aforementioned experimental data as, (i) residue Trp248 forms a cation-π interaction with the charged amide of V3 loop residue Arg18; (ii) the aromatic and hydroxyl groups of residue Tyr251 form polar and non-polar interactions with V3 loop residues Val19 and Trp20; and (iii) residue Leu255 forms strong non-polar contacts with V3 loop residue Trp20.

#### Role of the Extracellular Loop 3 and Transmembrane Helix 7 of CCR5

Experiments showed that an alanine mutation at Phe264 results in approximately a half loss of HIV-1 coreceptor activity [23], and in accordance with this, our complex structure shows that the aromatic group of Phe264 forms non-polar contacts with V3 loop residues Thr8 and Lys10. The same study showed that an alanine mutation to CCR5 residues Cys269 and to a smaller extent Phe262 has also a negative impact on the HIV-1 binding. According to both our study and the X-ray structure [38], Phe262 is not part of the binding site and points toward the exterior of TH6, while residue Cys269 forms a disulfide bridge with Cys20, and an alanine mutation at position 269 could be harmful for the structural integrity of CCR5. Furthermore, a study showed that the double Asp11Ala and Asp276Ala mutant of CCR5 reduces HIV-1 activity by 20–40% [26]; apart from the critical role of Asp11, our computationally derived structure also provides evidence for the role of Asp276 which forms polar and non-polar interactions with V3 loop residues Tyr21 and Thr22. Moreover, an additional experimental study showed that an alanine mutation on CCR5 residue Gln280 reduces significantly the HIV-1 activity, and this is in accordance with the results of our simulation as Gln280 is involved in important non-polar contacts with V3 loop residues Pro16, Val19 and Tyr21. Two studies confirmed the most important role of CCR5 Glu283 by showing that an alanine mutation abolishes HIV-1 binding, and a glutamine mutation decreases HIV-1 binding by approximately 75% [17], [18]. These findings suggest that while the non-polar moiety of Glu283 may partly play a role in binding, the negatively charged carboxyl group of Glu283 is utmost important for HIV-1 coreceptor activity. Our results provide compelling evidence for this as the charged carboxyl group of Glu283 forms a highly interacting salt bridge with V3 loop residue Arg18, and is also hydrogen bonded to the backbone amide of the same V3 loop residue; as a result, Glu283 and Arg18 are the most interacting residues of CCR5 and V3 loop, respectively. In addition, the non-polar side chain moiety of Glu283 forms contacts with V3 loop residues Gly17 and Val19. Also, a Met276Glu mutation at CCR5 results in an approximately 75% decrease of HIV-1 binding [18]; as Met276 is in the vicinity of V3 loop residue Arg18, it is possible that such a mutation would disorient the positively charged Arg18 side chain from binding to residue Glu283, and result in a not favorable V3 loop binding to CCR5.

We consider that our success in having exceptional agreement with, as well as, interpreting experimental results is due to the systematic methodology employed which includes the following features: (i) the modeling of the entire CCR5 structure and the use of an extensive set of computational tools and methods to produce a variety of structural templates of V3 loop and CCR5 for docking; (ii) the large number of docked complexes investigated; (iii) the heterogeneous dielectric solvation GB and PB models used to rank the complex structures according to their binding free energy; (iv) the employment of MD simulations for the most promising complexes, with regard to their binding free energy, in order to improve the conformational sampling and interactions, and (v) the selection of the final complex acquiring the lowest average binding free energy affinity throughout the MD simulations. Altogether, these steps constitute a systematic methodology, which we also recently applied to elucidate the molecular recognition of CXCR4 by the same dual tropic HIV-1 gp120 V3 loop [30]. A similar computational protocol has also recently proved its power in delineating the complex structure of CXCL12 (SDF-1α) in complex with CXCR4, in remarkable agreement with previous experimental findings [57]. The success of this method suggests its future application in elucidating additional chemokine : chemokine receptor complexes, or more generally, a broader series of ligand : membrane protein complexes.

While, the MM GBSA method accounts only for solvent entropy contributions and yields large binding free energy values, its use in the specific computational protocol proved advantageous, both here and in our recent study [30], with regard to identifying the lowest binding free energy, and thus, the optimum V3 loop : coreceptor simulated complex (see Table S1), which is in exceptional agreement with experiments. The simulated complexes with a low and comparable *ΔΔG* binding free energy with respect to the lowest binding free energy complex, Complex 14, have overall similar binding features, and their major differences are discussed in the *Information S1*. The simulated V3 loop : CCR5 system was modeled using the GBSW heterogeneous dielectric implicit water-membrane-water representation so as to accelerate the conformational sampling through an instantaneous conformational averaging over solvent and lipid molecules [68]. GBSW [52], IMM1 [69] and GBMV-HDGB [70], the three implicit membrane models available in CHARMM [54], have been widely used for the study of membrane peptides and proteins [54], and a recent study has suggested their potential use for membrane protein structure prediction [71]. It is worth noting that the dynamic HDGB (DHDGB) model is the latest advancement in implicit membrane models and allows a membrane deformation in response to the insertion of charged molecules, thereby avoiding the overestimation of insertion free energies with static membrane models [72].

### Insights into the Blocking Mechanism of HIV-1 gp120 by Maraviroc

The X-ray CCR5 structure in complex with maraviroc (PDB entry: 4MBS; resolution 2.7 Å) [38] lacks the N-terminal 1–18 domain which is critical for HIV-1 binding, and contains CCR5 segments 19 : 223, 227 : 313, with four engineered mutations: Cys58Tyr, Gly163Asn, Ala233Asp and Lys303Glu, and a rubredoxin fragment introduced between CCR5 residues 223 and 227. According to the crystal structure, the 19–31 N-terminal domain, the 181–188 region of ECL2 and the 301–313 C-terminal domain experience high flexibilities at least in the presence of maraviroc [38], and in the absence of the HIV-1 gp120 V3 loop. Tan *et al.* suggested that maraviroc inhibits chemokine function by blocking receptor activation through interactions within the transmembrane domain, and that maraviroc binding may reduce chemokine and gp120 binding in an allosteric inverse agonism manner by stabilizing CCR5 in an inactive conformation [38].

The exceptional agreement with experiments reported for the present computationally derived V3 loop : CCR5 structure provides us the capacity (i) to obtain insights into the identified and proper CCR5 conformation which is required for HIV-1 gp120 recognition, especially within the CCR5 - V3 loop binding - region, and in addition, (ii) to delineate how the interactions of maraviroc in complex with CCR5 interfere with the HIV-1 gp120 binding to CCR5.

The backbone RMSD between the CCR5 conformation bound to maraviroc (chain A) and the CCR5 conformation bound to V3 loop of the first simulation frame, within the transmembrane helical region comprising residues (28∶39, 82∶89, 98∶112, 159∶166, 189∶200, 252∶262, 275∶286) is 2.3 Å; the corresponding RMSD value reaches a plateau≈2.6 Å within the simulation. Thus, the relatively small value suggests that the transmembrane backbone conformation of CCR5 within the V3 loop and maraviroc binding site is similar, and the differences can mainly be attributed to different structural relaxation modes of CCR5 when it is bound to the HIV-1 gp120 V3 loop versus maraviroc. On the contrary, and based on the same superposition, the backbone RMSD between the CCR5 in our simulation and in complex with maraviroc, reaches a plateau of ≈4.9 Å for the N-terminal domain 19:27, and ≈5.0 Å for all ECL2 residues. This result is most presumably a consequence of the fact that the HIV-1 through its V3 loop can introduce conformational changes to the CCR5 N-terminal and the ECL2 upon binding. We observe that within the simulation, the N-terminal and the ECL2 domains of CCR5 loop are “locked” in relatively stable conformations, and their positions and relative orientations are well–defined and suitable for optimizing the coreceptor interactions with the V3 loop. Furthermore, the RMSD between the total transmembrane backbone of CCR5 bound to the V3 loop and the CCR5 bound to maraviroc for the entire transmembrane helical domain is ≈2.8 Å, and the larger contribution to this is attributed to residues 140∶157 in transmembrane helix 4. Thus, it is possible that maraviroc binding could also induce specific allosteric changes to specific transmembrane helical regions, which may be associated with an inactive CCR5 conformation, as suggested in [17], [38].

According to the X-ray structure of maraviroc in complex with CCR5 [38], the amine moiety of maraviroc's triazole group is hydrogen bonded to the hydroxyl group of CCR5 Tyr37, and also forms contacts with CCR5 residues Trp86 and Tyr89. The charged nitrogen of the tropane group forms a salt bridge with CCR5 residue Glu283, as well as hydrophobic contacts with CCR5 residue Tyr108. The phenyl group is surrounded by CCR5 aromatic/hydrophobic residues Tyr108, Phe109, Phe112, Ile198, Trp248 and Tyr251. The cyclohexane group is proximal to Phe182, Ile191 and Gln194, and forms hydrogen bonds with the hydroxyl groups of Thr195, Thr259 and the backbone carbonyl of Lys191, through its fluorine atoms. The intermolecular interactions of the maraviroc : CCR5 complex [38] are shown in Figure S3A. The HIV-1 gp120 V3 loop : CCR5 complex structure of this study reveals that maraviroc blocks the HIV-1 gp120 entry to CCR5 as it evidently interferes with the interactions between the majority of the aforementioned CCR5 residues and HIV-1 gp120 residues within the 12–20 V3 loop residue moiety (see Figures S3B,C). In general, maraviroc blocks the key transmembrane CCR5 region where the core of the V3 loop primarily binds (see Figure S3C). Specifically, it interferes with the formation of (i) the most highly interacting salt bridge between the V3 loop residue Arg18 and CCR5 residue Glu283, (ii) the hydrogen bonds between V3 loop Arg18 and CCR5 Tyr37, Tyr108, as well as the hydrogen bond between V3 loop residue Trp20 and CCR5 residue Tyr251, and (iii) a series of important non-polar interactions involving V3 loop residues 12–20 and CCR5 residues Tyr37, Trp86, Tyr89, Tyr108, Phe109, Lys191, Thr195, Ile198, Tyr251, Trp248, Thr259 and Glu283.

### Insights into the HIV-1 Coreceptor Selectivity

A recent study investigating the V3 loop tropism indicated that the regions informative of tropism belong to opposite stem regions [73]. Also, a subsequent recent study [74], which encompassed previously published knowledge on V3 loop tropism with regard to the “11/24/25” rule [41] and the presence of a specific glycosylation motif [75], verified the significance of charge as an additional key determinant to determine and increase the accuracy of coreceptor selectivity [74].

The HIV-1 gp120 V3 loop : CCR5 (presented here) and the HIV-1 gp120 V3 loop : CXCR4 [30] complex structures are in exceptional agreement with previous experimental findings, and thus, they represent excellent starting points for the study of a series of HIV-1 gp120 V3 loops in complex with CCR5/CXCR4, so as to obtain insights into the HIV-1 coreceptor selectivity. As a preliminary step toward this direction, we used CD-HIT [76] and extracted the eight most populated sequence-based clusters of - only CCR5 - and - only CXCR4 - recognizing 35-residue V3 loops from the Los Alamos HIV-1 database (http://www.hiv.lanl.gov), respectively. Based on the extracted sequences, we modeled and simulated eight - only CCR5 recognizing - HIV-1 gp120 V3 loop : CCR5 complexes, as well as eight - only CXCR4 recognizing - HIV-1 gp120 V3 loop : CXCR4 complexes (see *Information S3*). Upon the completion of the MD simulations and the extraction of simulation snapshots every 200 ps, we calculated the total interaction free energy of every V3 loop (1–35) residue position within the simulations for each of the complexes separately. Subsequently we evaluated the interaction free energy average and standard deviation values for every V3 loop (1–35) residue position in complex with CCR5 or CXCR4, independently. The results reveal that the key differences with regard to CCR5 versus CXCR4 binding correspond mainly to regions in opposite stem regions, in line with [73]; the same result was also deduced for the specific dual tropic V3 loop investigated in this study, in complex with CCR5/CXCR4 [30] (see Figure 3). We identified that V3 loop residue positions 3, 6, 7, 8, 11, 14, 15, 20 and 29 experience the largest (>4.5 kcal/mol) interaction free energy difference with regard to CCR5 versus CXCR4 binding; residue positions acquiring a high relative standard deviation (%RSD) were considered statistically insignificant and are not reported. Interestingly, among the aforementioned V3 loop residue positions, residue position 11 was also deemed important with regard to coreceptor selectivity in the “11/24/25” rule [41], and in addition, residue positions 6, 7, 8 were also deemed important with regard to coreceptor selectivity in the rule associated with the presence of a specific glycosylation motif [75]. While these preliminary results represent the first complex structure-based insights into coreceptor selectivity, a future systematic study, which will focus and examine in detail the specific interactions formed by V3 loop : coreceptor residue pairs in a larger series of CCR5/CXCR4/dual recognizing V3 loops : coreceptor complexes, will provide invaluable insights into coreceptor selectivity.

### Insights into the Design of Novel Peptides Targeting both CCR5 and CXCR4

As the dual tropic V3 loop which was used in this study to derive the present complete HIV-1 gp120 V3 loop : CCR5 complex structure is identical to the V3 loop used also to derive HIV-1 gp120 V3 loop : CXR4 complex structure [30], a comparison between the V3 loop bound conformations and the interaction free energies for every V3 loop residue in complex with CCR5/CXCR4 is of utmost importance for paving the way toward the design of a new generation of potential anti-AIDS therapeutics which can target both coreceptors.

Our results show that the HIV-1 gp120 V3 loop bound conformation is well defined and tight, at least for the specific dual tropic V3 loop sequence in complex with both CXCR4 [30] and CCR5 (in the present work), and the bound V3 loop adopts a maximized tip-base conformation, one of the key unbound V3 loop conformations identified in [31]. By comparing the binding of the specific dual tropic HIV-1 gp120 V3 loop with CCR5 and CXCR4 [30], we observe that the HIV-1 gp120 V3 loop residues 13–21, which include the tip, share nearly identical structural and energetic properties in complex with both coreceptors. This finding suggests that novel peptides can be designed to mimic the bound conformation and energetic properties of the specific dual tropic V3 loop 13–21 domain, in complex with CXCR4 [30] and CCR5, aiming at producing compounds which can simultaneously target both CCR5 and CXCR4.

## Conclusions

We report, what is to our knowledge, the first complete HIV-1 gp120 V3 loop : CCR5 complex structure which exhibits exceptional agreement with previous experimental findings. Previous studies which aimed at investigating the same problem, and either considered the entire CCR5 protein [19], [36], [37], [39] or not [8], [38], have not reported a high-degree of agreement with regard to a wide spectrum of experimental findings. Apart from shedding light into the functional role of HIV-1 gp120 and CCR5 residues related to coreceptor activity, this study (i) provides insights into HIV-1 coreceptor selectivity, (ii) reveals how maraviroc interferes with the entry of HIV-1 gp120 protein into CCR5, through a direct comparison of our derived structure to the recent X-ray structure of CCR5 in complex with maraviroc [38], and (iii) provides a detailed interaction free energy based comparison between the HIV-1 gp120 V3 loop residues of the specific dual tropic V3 loop in complex with CCR5 versus CXCR4 [30]. Our results reveal that the key interacting differences, with regard to binding of the specific V3 loop to the chemokine receptors CCR5/CXCR4 [30], are associated with V3 loop residues primarily out of the 13–21 residue moiety, and that V3 loop residues 13–21 share nearly identical structural and energetic properties in complex with both coreceptors. This finding paves the way for the discovery of a peptide compound which can target both CCR5 and CXCR4. The *de novo* design [60], [62], [77]–[82] of peptide compounds which would mimic the bound conformation and energetic properties of the specific dual tropic HIV-1 gp120 V3 loop 13–21 domain, in complex with both CCR5 and CXCR4 [30], is a primary future direction.

## Supporting Information

Information S1 **Comparison of Complexes 1, 3, 6, 12 to Complex 14.**
(DOCX)

Information S2 **Supporting Coordinates are provided in PDB format.**
(DOCX)

Information S3 **Insights into the HIV-1 Coreceptor Selectivity.**
(DOCX)

Information S4 **Definition of CCR5 and V3 loop domains.**
(DOCX)

Figure S1 **A) V3 loop : V3 Loop Residue Pairwise Intramolecular Interaction Free Energies. B) Buried Surface Area of V3 loop Residues.**
(DOCX)

Figure S2 **V3 loop : CCR5 Residue Pairwise Intermolecular Interaction Free Energies.**
(DOCX)

Figure S3 **Maraviroc versus the HIV-1 gp120 V3 loop binding to CCR5.**
(DOCX)

Table S1 **Binding free energies using the PBSA, MM GBSA and MM PBSA approximations for V3 loop : CCR5 complexes.**
(DOCX)

Table S2 **Average and standard deviation RMSD of the simulation coordinates with respect to the corresponding coordinates from the first simulation frames after equilibration.**
(DOCX)

Table S3 **Hydrogen bond percentage (%) occupancies of important intermolecular hydrogen-bonding atom pairs within Complexes 1, 3, 6, 12, 14.**
(DOCX)

Video S1 **A video demonstrating the simulation trajectory of V3 loop : CCR5 in Complex 14, and depicting the key salt bridges and specific important hydrogen bonds is provided.**
(ZIP)

Coordinates S1 (PDB)

Coordinates S2 (PDB)

Coordinates S3 (PDB)

Coordinates S4 (PDB)

Coordinates S5 (PDB)

Coordinates S6 (PDB)

Coordinates S7 (PDB)

Coordinates S8 (PDB)

Coordinates S9 (PDB)

Coordinates S10 (PDB)
