# Supplementary material for: Molecular Recognition of CCR5 by an HIV-1 gp120 V3 Loop
Source: PLoS One. 2014 Apr 24;9(4):e95767. doi: 10.1371/journal.pone.0095767 (PMC3999033; doi:10.1371/journal.pone.0095767)
Supplement: Information S1 — Comparison of Complexes 1, 3, 6, 12 to Complex 14. (DOCX) [file pone.0095767.s001.docx]

***Information S1***

***Comparison of Complexes 1, 3, 6, 12 to Complex 14:***

Complex 14 possesses the most favorable binding free energy as it evidently acquires the lowest combination of non-polar and polar binding free energies according to the MM GBSA approximation. As the MM GBSA approximation provides an appropriate and clear separation between the top ranked Complex and the rest, we use its results as our metric for our comparative analysis. The top ranked Complexes, 1, 3, 6, 12 and 14, differ mainly with regard to the polar binding free energy component, rather than the non-polar component. This predominantly occurs owing to the variabilities in polar V3 loop : CCR5 interactions, which are primarily related to intermolecular salt bridges, as they capture the largest portion of the polar component.

In all complexes, the Arg18 : Glu283 salt bridge is maintained throughout the simulations. Complexes 6, 12 and 14 possess the lowest polar binding free energy components, and this can be attributed to the fact that they encompass the following same V3 loop : CCR5 salt bridge residue pairs: Arg9 : Asp2, Arg18 : Glu283, Arg31:Asp11, Lys10 : Glu262 (in Complexes 6, 12), Arg11 : Glu172 (in Complexes 12, 14), Arg29: charged N-terminal of Met1 (in Complexes 6, 14), Arg31 : Tys14 (in Complexes 12, 14). The key salt bridges which are uniquely present in only one of the three complexes are: Arg3 : Asp11, Arg9 : Tys10 in Complex 6, Arg3 : Glu18 in Complex 12, and Lys32 : Asp11 in Complex 14. On the other hand, Complexes 1 and 3, which acquire somewhat less favorable polar binding free energy component with regard to the three top ranked complexes, they mutually share similar salt bridges which involve the V3 loop : CCR5 residue pairs: Arg3 : Tys14, Arg9 : Asp11, Lys10 : Asp11, Arg11 : Glu172, Arg18 : Glu283, Arg31 : Tys3. Complex 3 is more energetically favored than Complex 1 due to the additional salt bridge Lys32 : Asp11. This salt bridge is also present in Complex 14, and interestingly, owing to this, Complex 3 is energetically more favored than Complex 1, and similarly Complex 14 is more energetically favored than Complexes 6 and 12.

The specificity of the salt bridges between V3 loop residue : CCR5 residue, their occupancy, as well as the dielectric medium at which they occur determines their degree of contribution with regard to the polar binding free energy for each complex separately. Overall, the polar intermolecular interactions are optimum in Complex 14 compared to the rest complexes, and owing to this, Complex 14 is according to the MM GBSA approximation the top ranked complex.
