# Supplementary material for: Molecular Recognition of CCR5 by an HIV-1 gp120 V3 Loop
Source: PLoS One. 2014 Apr 24;9(4):e95767. doi: 10.1371/journal.pone.0095767 (PMC3999033; doi:10.1371/journal.pone.0095767)
Supplement: Information S2 — Supporting Coordinates are provided in PDB format. (DOCX) [file pone.0095767.s002.docx]

***Information S2***

***Supporting Coordinates are provided in PDB format:***

The MD coordinates of Complex 14, extracted every 2 ns, are provided as Supporting Information in PDB format. The structures are aligned with regard to the backbone of the CCR5 transmembrane helical region. The correspondence of PDB files and time in the MD simulation is as follows:

| PDB file | Time (ns) |
| --- | --- |
| coordinates.s1.pdb | 2 |
| coordinates.s2.pdb | 4 |
| coordinates.s3.pdb | 6 |
| coordinates.s4.pdb | 8 |
| coordinates.s5.pdb | 10 |
| coordinates.s6.pdb | 12 |
| coordinates.s7.pdb | 14 |
| coordinates.s8.pdb | 16 |
| coordinates.s9.pdb | 18 |
| coordinates.s10.pdb | 20 |
