# Supplementary material for: Molecular Recognition of CCR5 by an HIV-1 gp120 V3 Loop
Source: PLoS One. 2014 Apr 24;9(4):e95767. doi: 10.1371/journal.pone.0095767 (PMC3999033; doi:10.1371/journal.pone.0095767)
Supplement: Information S3 — Insights into the HIV-1 Coreceptor Selectivity. (DOCX) [file pone.0095767.s003.docx]

***Information S3***

***Insights into the HIV-1 Coreceptor Selectivity:***

We used CD-HIT^[[1]](#endnote-1)^ to extract the eight most populated sequence-based clusters of - only CCR5 - and - only CXCR4 - recognizing 35-residue V3 loops, respectively, from the Los Alamos HIV-1 database (<http://www.hiv.lanl.gov>).

| CCR5  Cluster | CCR5 recognizing V3 loop sequences; the eight most populated 35-residue sequences were selected | CXCR4  Cluster | CXCR4 recognizing V3 loop sequences; the eight most populated 35-residue sequences were selected |
| --- | --- | --- | --- |
| 1 | CPRPNNNTRKSVRIGPGQTFYATGDIIGDIRQAHC | 1 | CTRPYNNTRTSVRIGPGQAFYKTGEIVGDIRRAYC |
| 2 | CTRPNNNTRKSIPIGPGRAFYTTGEIIGDIRKAHC | 2 | CTRPNNNTRRRIYIGQGRAVYTTKQIVGDIRKAYC |
| 3 | CTRPNNNTRKSIPMGPGKAFYATGDIIGDIRQAHC | 3 | CTRPFKNIRTSVRIGPGRVFYKTGAITGDIRRAYC |
| 4 | CTRPNNNTRKSIHIGPGRAFYTTGQIIGDIRQAYC | 4 | CTRPNNNTRQRISIGPGRAFYTTRQVIGDIRQAHC |
| 5 | CIRPNNNTRKSIRIGPGQAFYATGDIIGDIRQAYC | 5 | CTRPSNPTRTRITMGPGRVWYRTGEITGSIRKAYC |
| 6 | CTRPNNNTRKGIHIGPGRTFYTTGEIIGDIRQAHC | 6 | CTRPFNNTRTSVGVGPGQVFYKTGDIIGDIRRAYC |
| 7 | CTRPNNNTRRSINIGPGRAFYTTGDIIGDIRQAHC | 7 | CIRPNNNTRKSISFGPGQAFYATGDIIGDIRQAYC |
| 8 | CTRPNNNTRKSISIGPGRAFYATGEIIGDIRQAYC | 8 | CARPFYAIERQRTPIGQGQVLYTTKKIGRIGQAHC |

We used the starting conformations of Complex 14 in the present study, and Complex 1 from the molecular recognition of CXCR4 by the same dual tropic V3 loop, by Tamamis and Floudas^[[2]](#endnote-2)^, to construct the new complex structures with the appropriate mutations, and perform MD simulations of clustered CCR5 recognizing and CXCR4 recognizing V3 loops; V3 loop sequences which did not have a sequence length of 35 residues and were excluded from this analysis. The MD simulations consisted of two parts: (i) sampling, and (ii) production. In the sampling part, the starting conformations in all twenty complexes were simulated for 4 ns using the exact same protocol of step 5 in Methods. Structures were extracted every 20 ps, and the lowest binding free energy structure was selected, in each complex, as a new starting point for the conduction of the production run which consisted of a light equilibration and 5 ns of production run. Upon the completion of the MD simulations and the extraction of simulation snapshots every 200 ps in the production run, in each complex, we calculated the total interaction free energy of every V3 loop (1-35) residue position within the simulations for each of the complexes separately; 25 snapshots were analyzed for each Complex. Subsequently we evaluated the interaction free energy average and standard deviation values for every V3 loop (1-35) residue position in complex with CCR5 or CXCR4 independently (see *Discussion* in main text). The parametrization and setup of MD simulations is identical to the one used in Step 5 of *Methods*.

1. Weizhong L, Godzik A. (2006) Cd-hit: a fast program for clustering and comparing large sets of protein or nucleotide sequences. Bioinformatics 22:1658-1659. [↑](#endnote-ref-1)
2. Tamamis P, Floudas CA. (2013) Molecular recognition of CXCR4 by a dual tropic HIV-1 gp120 V3 loop. Biophys J 105(6): 1502-1514. [↑](#endnote-ref-2)
