# Supplementary material for: Molecular Recognition of CCR5 by an HIV-1 gp120 V3 Loop
Source: PLoS One. 2014 Apr 24;9(4):e95767. doi: 10.1371/journal.pone.0095767 (PMC3999033; doi:10.1371/journal.pone.0095767)
Supplement: Information S4 — Definition of CCR5 and V3 loop domains. (DOCX) [file pone.0095767.s004.docx]

***Information S4***

***Definition of CCR5 and V3 loop domains:***

CCR5 residues, denoted in brackets, can be approximately divided into the following domains: (i) N-terminal domain [1:27], (ii) Transmembrane helix 1 (TH1) [28:59]; (iii) Intracellular loop 1 (ICL1) [60:65]; (iv) Transmembrane helix 2 (TH2) [66:89], (v) Extracellular loop 1 (ECL1) [90:97]; (vi) Transmembrane helix 3 (TH3) [98:132]; (vii) Intracellular loop 2 (ICL2) [133:139]; (viii) Transmembrane helix 4 (TH4) [140:166]; (ix) Extracellular loop 2 (ECL2) [167:188]; (x) Transmembrane helix (TH5) [189:219]; (xi) Intracellular loop 3 (ICL3) [220:231]; (xii) Transmembrane helix 6 (TH6) [232:262]; (xiii) Extracellular loop 3 (ECL3) [263:274]; (xiv) Transmembrane helix 7 (TH7) [275:301]; (xv) C-terminal domain [302:352]. Regarding the V3 loop, the structure can approximately be divided into the following regions, based on the V3 loop binding to CCR5: (i) The base domain [1:4 and 31:35]; (ii) Stems [5:12 and 23:30] (iii) Tip [13:22]. According to the results of this study, the core region of the tip comprises residues 16-20.
