# Supplementary material for: Molecular Recognition of CCR5 by an HIV-1 gp120 V3 Loop
Source: PLoS One. 2014 Apr 24;9(4):e95767. doi: 10.1371/journal.pone.0095767 (PMC3999033; doi:10.1371/journal.pone.0095767)
Supplement: Figure S1 — A) V3 loop : V3 Loop Residue Pairwise Intramolecular Interaction Free Energies. B) Buried Surface Area of V3 loop Residues. (DOCX) [file pone.0095767.s005.docx]

**Figure S1A: V3 loop : V3 Loop Residue Pairwise Intramolecular Interaction Free Energies.**


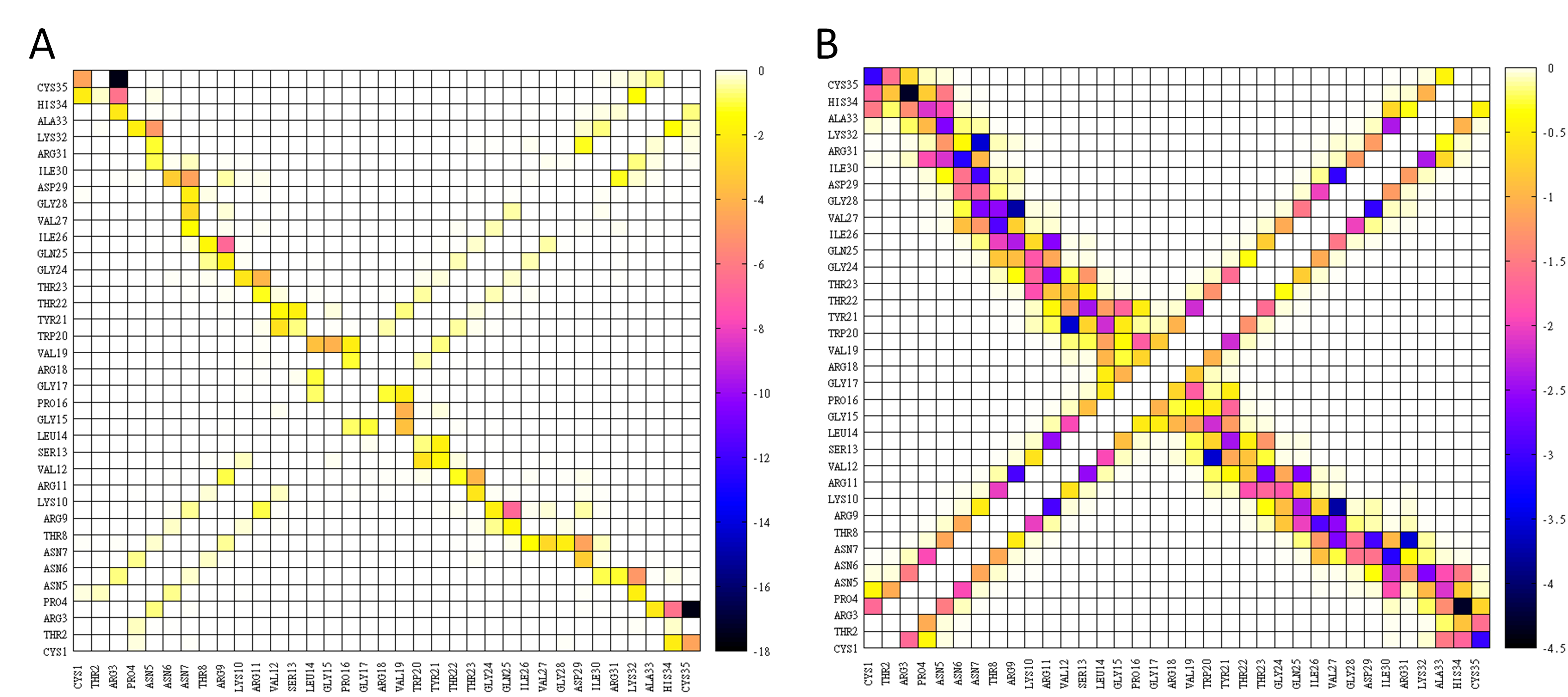


**Figure S1A:** Two dimensional density maps depicting the favorable (negative) average MM GBSA interaction free-energies for intramolecular V3 loop interacting residue pairs, within the simulation of the complex with the lowest average binding free energy. The left (A) and right (B) panels correspond to polar and non-polar interactions, respectively. All energies are in kcal/mol. The color – interaction free energy correspondence is shown by the palette on the right-hand side of each panel. Interaction free energies were not calculated for pairs of covalently bonded residues. All values have been computed by analysis of 1000 snapshots, extracted from the 20-ns simulation of complex 14, at 20-ps intervals. The analysis was performed using CHARMM^[[1]](#endnote-1)^ and in-house FORTRAN programs.

**Figure S1B: Buried Surface Area of V3 loop Residues.**


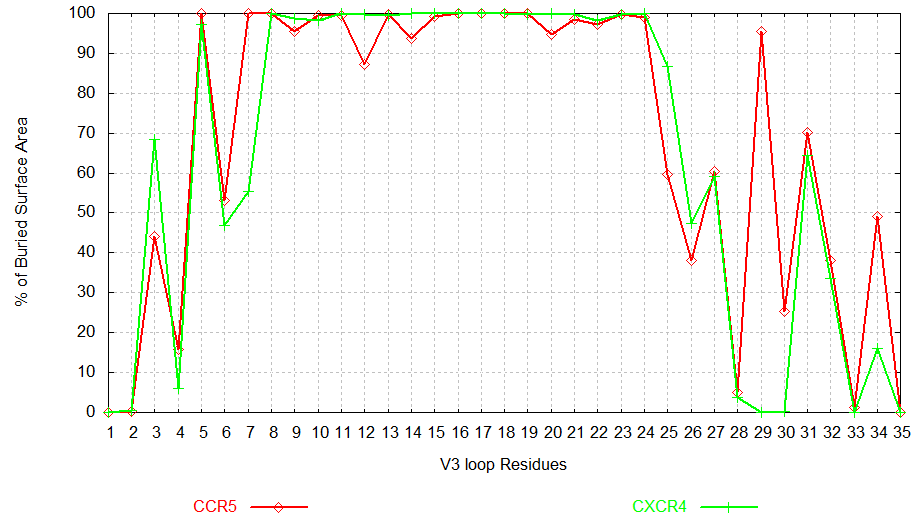


**Figure S1B:**

Average percentage (%) of buried surface area of each V3 loop residue in complex with CCR5 (red color) or CXCR4^[[2]](#endnote-2)^ (green color), normalized by the total surface accessible area of the corresponding residue in its unbound state. A value of 100 % shows that the specific residue is burying its total accessible surface area owing to contacts with the coreceptor, a value of 50 % shows that the specific residue is burying half of its total accessible surface area owing to contacts with the coreceptor, whereas value of 0% shows that the residue is not contacting the coreceptor. All values have been computed by analysis of 1000 snapshots, extracted from the 20-ns simulation of complex 14 (CCR5) and Complex 1 (CXCR4)^1^, at 20-ps intervals. The probe radius was universally set to 1.4 Å (for both the water and the membrane environment), as a similar value for the membrane environment (1.3 Å) was able to reproduce experimental results in a previous study^[[3]](#endnote-3)^, in contrast to larger values (2.2 or 4.8 Å). The analysis was performed using CHARMM^1^ and in-house FORTRAN programs.

1. . Brooks BR, Brooks CL III, Mackerell AD Jr, Nilsson L, Petrella RJ, Roux B, Won Y, Archontis G, Bartels C, Boresch S, Caflisch A, Caves L, Cui Q, Dinner AR Feig, M, Fischer S, Gao J, Hodoscek M, Im W, Kuczera K, Lazaridis T, Ma J, Ovchinnikov V, Paci E, Pastor RW, Post CB, Pu JZ, Schaefer M, Tidor B, Venable RM, Woodcock HL, Wu X, Yang W, York DM, Karplus MJ. (2009) CHARMM: the biomolecular simulation program. J Comput Chem 30: 1545–1614. [↑](#endnote-ref-1)
2. Tamamis P, Floudas CA. (2013) Molecular recognition of CXCR4 by a dual tropic HIV-1 gp120 V3 loop. Biophys J 105(6): 1502-1514. [↑](#endnote-ref-2)
3. . Woolf TB, Roux B. (1996) Structure, energetics, and dynamics of lipid-protein interactions: A molecular dynamics study of the gramicidin A channel in a DMPC bilayer. Proteins 24(1): 92-114. [↑](#endnote-ref-3)
