# Supplementary material for: Molecular Recognition of CCR5 by an HIV-1 gp120 V3 Loop
Source: PLoS One. 2014 Apr 24;9(4):e95767. doi: 10.1371/journal.pone.0095767 (PMC3999033; doi:10.1371/journal.pone.0095767)
Supplement: Figure S2 — V3 loop : CCR5 Residue Pairwise Intermolecular Interaction Free Energies. (DOCX) [file pone.0095767.s006.docx]

**Figure S2: V3 loop : CCR5 Residue Pairwise Intermolecular Interaction Free Energies.**


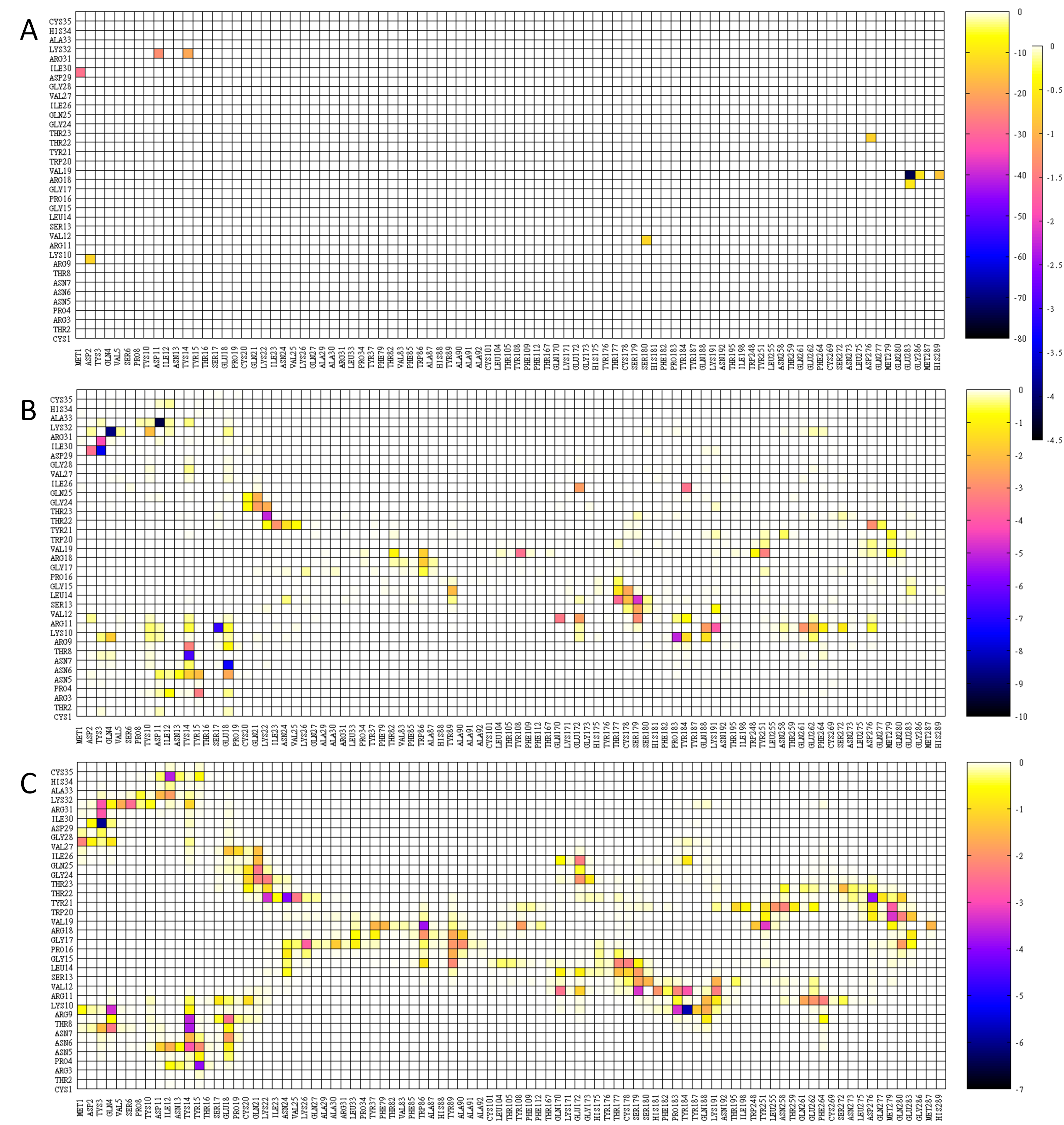


**Figure S2:** Two dimensional density maps depicting the favorable (negative) average MM GBSA interaction free-energies for intermolecular V3 loop (y-axis) : CCR5 (x-axis) interacting residue pairs, within the simulation of the complex with the lowest average binding free energy. The upper (A), middle (B) and bottom (C) panels correspond to highly interacting polar [-80 kcal/mol : -10 kcal/mol], moderate interacting polar [-10 kcal/mol : 0 kcal/mol], and non-polar interactions, respectively. All energies are in kcal/mol. The color – interaction free energy correspondence is shown by the palette on the right-hand side of each panel. All values have been computed by analysis of 1000 snapshots, extracted from the 20-ns simulation of complex 14, at 20-ps intervals. The analysis was performed using CHARMM^[[1]](#endnote-1)^ and in-house FORTRAN programs.

1. Brooks BR, Brooks CL III, Mackerell AD Jr, Nilsson L, Petrella RJ, Roux B, Won Y, Archontis G, Bartels C, Boresch S, Caflisch A, Caves L, Cui Q, Dinner AR Feig, M, Fischer S, Gao J, Hodoscek M, Im W, Kuczera K, Lazaridis T, Ma J, Ovchinnikov V, Paci E, Pastor RW, Post CB, Pu JZ, Schaefer M, Tidor B, Venable RM, Woodcock HL, Wu X, Yang W, York DM, Karplus MJ. (2009) CHARMM: the biomolecular simulation program. J Comput Chem 30: 1545–1614. [↑](#endnote-ref-1)
