# Supplementary material for: Molecular Recognition of CCR5 by an HIV-1 gp120 V3 Loop
Source: PLoS One. 2014 Apr 24;9(4):e95767. doi: 10.1371/journal.pone.0095767 (PMC3999033; doi:10.1371/journal.pone.0095767)
Supplement: Figure S3 — Maraviroc versus the HIV-1 gp120 V3 loop binding to CCR5. (DOCX) [file pone.0095767.s007.docx]

**Figure S3: Maraviroc versus the HIV-1 gp120 V3 loop binding to CCR5.**

**
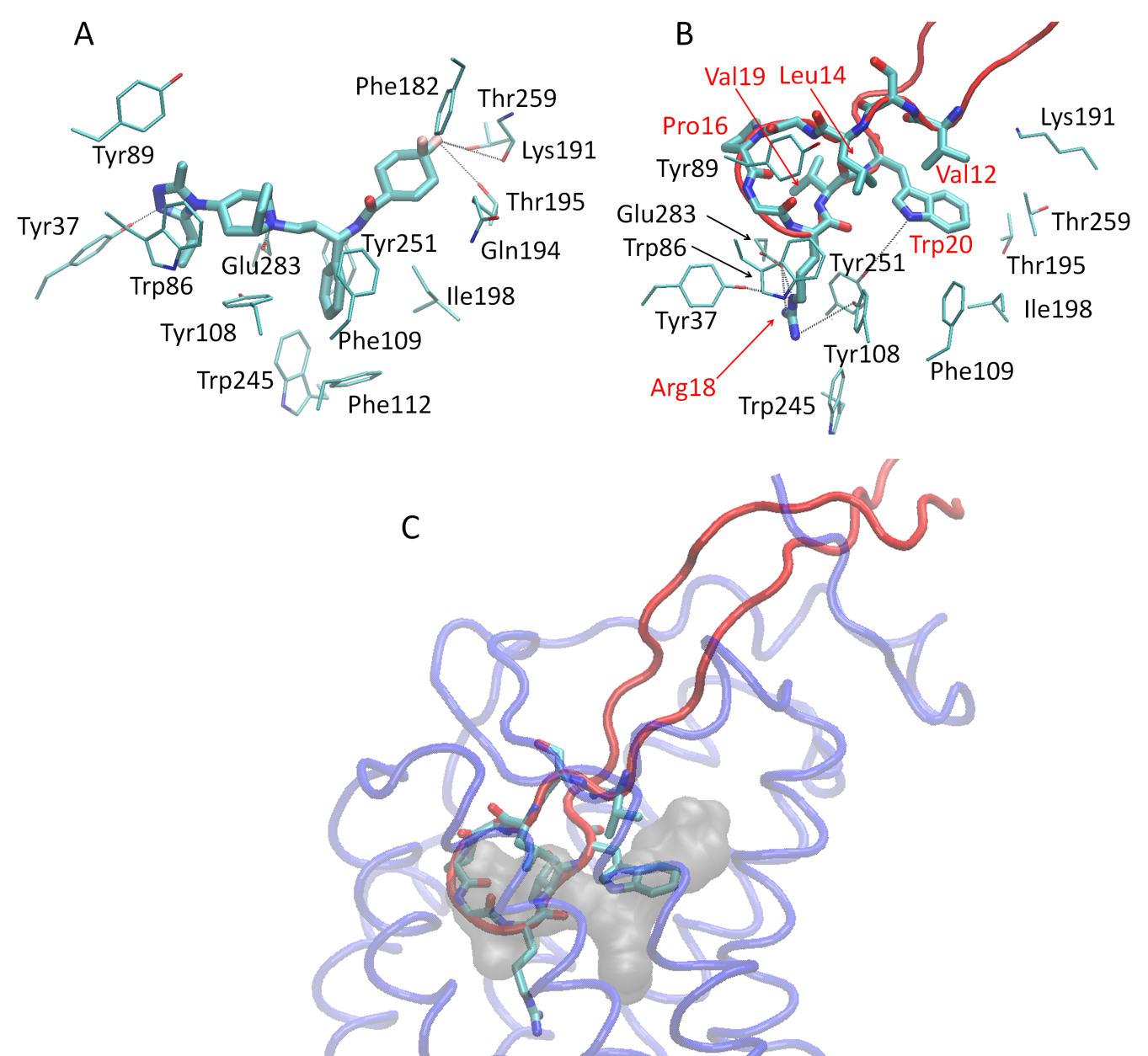
**

**Figure S3:** (A) Molecular graphics image showing how maraviroc binds to CCR5^[[1]](#endnote-1)^. Maraviroc is shown in thick licorice and CCR5 residues in thin licorice representation. (B) Molecular graphics image of V3 loop (in red tube representation) binding to CCR5, focusing at the binding site of maraviroc. Panel (B) presents the overlapping CCR5 residues with respect to panel (A), to denote which interactions between the V3 loop and CCR5 (B) are interfered in the presence of maraviroc (A). Salt bridges/Hydrogen bonds in (A) and (B) are shown in dashed lines. V3 loop residues are shown in thick licorice and CCR5 residues in thin licorice representation. (C) Molecular graphics image showing the binding site of maraviroc (in gray surface representation) blocking the 12-20 V3 loop residue moiety, after superposition of the two complex structures; the 12-20 V3 loop residues are shown in thick licorice. The V3 loop and CCR5 are shown in red and blue tube representation. The labels for CCR5 and V3 loop residues are annotated in in black and red color, respectively. Hydrogen atoms are omitted for clarity.

1. . Tan Q, Zhu Y, Li J, Chen Z, Han GW, Kufareva I, Li T, Ma L, Fenalti G, Li J, Zhang W, Xie X, Yang H, Jiang H, Cherezov V, Liu H, Stevens RC, Zhao Q, Wu B. (2013) Structure of the CCR5 Chemokine Receptor-HIV Entry Inhibitor Maraviroc Complex. Science 341: 1387:1390. [↑](#endnote-ref-1)
