# Supplementary material for: Molecular Recognition of CCR5 by an HIV-1 gp120 V3 Loop
Source: PLoS One. 2014 Apr 24;9(4):e95767. doi: 10.1371/journal.pone.0095767 (PMC3999033; doi:10.1371/journal.pone.0095767)
Supplement: Table S1 — Binding free energies using the PBSA, MM GBSA and MM PBSA approximations for V3 loop : CCR5 complexes. (DOCX) [file pone.0095767.s008.docx]

**Table S1:**

Binding free energies using the PBSA, MM GBSA and MM PBSA approximations for V3 loop : CCR5 complexes.

|  | Docking and Minimization^¶^ | | | MD Simulations^†^ | | | | |
| --- | --- | --- | --- | --- | --- | --- | --- | --- |
|  | PBSA | | | MM GB/PBSA (Non-Polar)^‡^ | MM GBSA^ǂ^ (Polar and Total) | | MM PBSA^#^ (Polar and Total) | |
|  | Non-Polar | Polar | Total | Non- polar | Polar | Total | Polar | Total |
| 1^§^ | -177.4 | -43.3 | -220.7 | -289.8 (13.9) | -85.1 (15.4) | -374.8 (13.2) | -62.3 (13.1) | -352.1 (11.9) |
| 2 | -180.1 | -32.5 | -212.6 | -264.4 (8.7) | -60.6 (17.9) | -324.9 (18.7) | - | - |
| 3 | -168.8 | -41.6 | -210.4 | -288.6 (12.8) | -101.6 (13.8) | -390.2 (15.0) | -32.9 (14.1) | -321.5 (14.5) |
| 4 | -180.8 | -28.7 | -209.5 | -262.6 (9.0) | -77.1 (13.6) | -339.8 (14.7) | - | - |
| 5 | -157.6 | -48.2 | -205.8 | -264.5 (10.9) | -93.3 (15.8) | -357.9 (18.0) | - | - |
| 6 | -165.9 | -39.8 | -205.6 | -281.8 (11.4) | -114.9 (15.6) | -396.7 (18.5) | -70.0 (10.7) | -351.8 (14.8) |
| 7 | -170.5 | -31.4 | -201.9 | -267.6 (12.6) | -69.0 (13.1) | -336.6 (15.2) | - | - |
| 8 | -174.7 | -26.3 | -201.0 | -266.8 (9.7) | -111.4 (17.7) | -378.2 (15.7) | -71.7 (14.1) | -338.5 (12.7) |
| 9 | -186.7 | -12.7 | -199.5 | -284.0 (15.1) | -57.4 (11.0) | -341.4 (14.6) | - | - |
| 10 | -192.1 | -7.2 | -199.4 | -283.7 (9.0) | -54.2 (11.8) | -337.9 (11.4) | - | - |
| 11 | -167.7 | -30.3 | -198.0 | -255.5 (10.2) | -84.0 (14.1) | -339.5 (12.2) | - | - |
| 12 | -186.0 | -12.0 | -197.9 | -275.6 (9.7) | -119.1 (12.2) | -394.8 (11.1) | -77.8 (8.5) | -353.4 (9.1) |
| 13 | -164.8 | -32.3 | -197.0 | -275.3 (9.7) | -99.4 (11.9) | -374.7 (14.2) | -46.6 (8.7) | -321.9 (11.9) |
| 14 | -184.5 | -12.4 | -196.8 | -278.5 (9.8) | -140.0 (14.7) | **-418.5 (15.2)** | -71.5 (11.1) | -350.1 (10.9) |
| 15 | -161.5 | -35.0 | -196.5 | -290.2 (11.8) | -82.5 (14.8) | -372.7 (14.5) | -52.8 (10.3) | -342.9 (12.8) |
| 16 | -207.3 | 11.6 | -195.7 | -252.1 (9.2) | -85.3 (13.0) | -337.3 (11.2) | - | - |
| 17 | -169.5 | -26.1 | -195.6 | -288.6 (13.0) | -94.5 (15.1) | -383.1 (16.4) | -28.1 (14.4) | -316.7 (12.4) |
| 18 | -186.8 | -8.4 | -195.2 | -288.1 (11.0) | -89.4 (13.2) | -377.5 (17.4) | -35.2 (11.5) | -323.3 (13.2) |
| 19 | -189.5 | -5.6 | -195.1 | -270.6 (9.7) | -66.9 (11.5) | -337.4 (12.3) | - | - |

The binding free energies (kcal/mol) are calculated as described in Methods. The numbering^§^ (1-19) is sorted according to the total binding free energy, using the PBSA approximation, of the docked complexes after minimization, referred as step 4^¶^; the total binding free energy is the sum of non-polar and polar contributions of step 4. The MD simulation^†^ results correspond to the average binding free energies of the 19 complexes based on 20-ns MD simulation runs, referred as step 6, and were calculated both using MM GBSA ^ǂ^  and MM PBSA^#^. The standard deviation is shown in parentheses. The non-polar component^‡^ is the same in MM GBSA^ǂ^ and MM GBSA^#^. For each calculation, MM GBSA^ǂ^ or MM PBSA^#^, the polar component is calculated using a different approach, thus, the total binding free energy, which is the sum of polar and non-polar contributions for each approach is different, as well. MM PBSA^#^ was applied to Complex 14, and additionally to Complexes 1, 3, 6, 8, 12, 13, 14, 15, 17, 18 only, as these complexes possessed a binding free energy within a (ΔΔG) range of 50 kcal/mol of Complex14, with regard to MM GBSA^ǂ^. According to MM GBSA^ǂ^, the average total binding free energy of the Complex 14, marked in **bold** face, is the lowest, and is by ≈22 and ≈24 kcal/mol (ΔΔG) lower compared to Complexes 6 and 12, respectively. According to MM PBSA^#^, the binding free energy of Complex 14 is comparable to the binding free energies of Complexes 1, 6 and 12. All values have been computed by analysis of 1000 snapshots, extracted from the 20-ns simulation (of all complexes), at 20-ps intervals. The analysis was performed using CHARMM^[[1]](#endnote-1)^ and in-house FORTRAN programs.

1. . Brooks BR, Brooks CL III, Mackerell AD Jr, Nilsson L, Petrella RJ, Roux B, Won Y, Archontis G, Bartels C, Boresch S, Caflisch A, Caves L, Cui Q, Dinner AR Feig, M, Fischer S, Gao J, Hodoscek M, Im W, Kuczera K, Lazaridis T, Ma J, Ovchinnikov V, Paci E, Pastor RW, Post CB, Pu JZ, Schaefer M, Tidor B, Venable RM, Woodcock HL, Wu X, Yang W, York DM, Karplus MJ. (2009) CHARMM: the biomolecular simulation program. J Comput Chem 30: 1545–1614. [↑](#endnote-ref-1)
