# Supplementary material for: Molecular Recognition of CCR5 by an HIV-1 gp120 V3 Loop
Source: PLoS One. 2014 Apr 24;9(4):e95767. doi: 10.1371/journal.pone.0095767 (PMC3999033; doi:10.1371/journal.pone.0095767)
Supplement: Table S2 — Average and standard deviation RMSD of the simulation coordinates with respect to the corresponding coordinates from the first simulation frames after equilibration. (DOCX) [file pone.0095767.s009.docx]

**Table S2:**

Average and standard deviation RMSD of the simulation coordinates with respect to the corresponding coordinates from the first simulation frames after equilibration.

| Complex | Protein Transmembrane Region | | Protein N-terminal, residues 1 -27 | | V3 loop | | V3 loop, residues: 8-26 | |
| --- | --- | --- | --- | --- | --- | --- | --- | --- |
|  | Average | St. Deviation | Average | St. Deviation | Average | St. Deviation | Average | St. Deviation |
| 1 | 1.23 | 0.05 | 3.41 | 0.16 | 5.39 | 1.11 | 1.83 | 0.04 |
| 3 | 1.10 | 0.03 | 2.85 | 0.30 | 2.98 | 1.08 | 1.55 | 0.13 |
| 6 | 1.24 | 0.05 | 6.04 | 1.60 | 6.76 | 3.26 | 2.18 | 0.28 |
| 12 | 1.05 | 0.02 | 2.91 | 0.29 | 3.30 | 0.47 | 1.14 | 0.03 |
| 14 | 1.14 | 0.01 | 2.68 | 0.09 | 2.06 | 0.11 | 1.35 | 0.05 |

The coordinates of the simulated systems are aligned (superimposed) with regard to the transmembrane backbone CCR5 atoms of the first simulation frames in each complex. All values corresponding to protein N-terminal, V3 loop and V3 loop residues 8-26, are computed without any additional rotation/translation and are averaged over the 20 ns of the simulation trajectories. Complexes 1, 3, 6, 12, 14 were considered in the analysis as they are the top five ranked complexes according to MM GBSA and MM PBSA. All values have been computed by analysis of 1000 snapshots (per complex), extracted from the 20-ns simulations, at 20-ps intervals. All values are reported in Å. Only the backbone heavy atoms N, Cα, and C were considered in the RMSD calculations.
