# Supplementary material for: Molecular Recognition of CCR5 by an HIV-1 gp120 V3 Loop
Source: PLoS One. 2014 Apr 24;9(4):e95767. doi: 10.1371/journal.pone.0095767 (PMC3999033; doi:10.1371/journal.pone.0095767)
Supplement: Table S3 — Hydrogen bond percentage (%) occupancies of important intermolecular hydrogen-bonding atom pairs within Complexes 1, 3, 6, 12, 14. (DOCX) [file pone.0095767.s010.docx]

**Table S3:**

Hydrogen bond percentage (%) occupancies of important intermolecular hydrogen-bonding atom pairs within Complexes 1, 3, 6, 12, 14. These complexes were analyzed as they are the top five ranked complexes according to MM GBSA and MM PBSA. Hydrogen bonding atom pairs, acquiring an occupancy of less than 10% are not reported. We present the hydrogen bond occupancies for each complex in separate tables and sort the hydrogen bonding atom pairs, firstly, with respect to the residue number of the V3 loop atom, and secondly, the residue number of the CCR5 atom. All values have been computed by analysis of 1000 snapshots (per complex), extracted from the 20-ns simulations, at 20-ps intervals. A hydrogen bond was present if the donor (D)–acceptor (A) distance was less than 3.5 Å, and the corresponding angle (D–H ^…^ A) was larger than 90°. Hydrogen bond interactions associated with salt-bridge formation are highlighted.

Complex 1:

| V3 loop Residue | Number | Group | Atom | CCR5 Residue | Number | Group | Atom | Occupancy (%) |
| --- | --- | --- | --- | --- | --- | --- | --- | --- |
| ARG | 3 | Side | NH1 | TYS | 14 | Side | OS4 | 32.4 |
| ARG | 3 | Side | NE | TYS | 14 | Side | OS4 | 18.2 |
| ARG | 3 | Side | NH1 | TYR | 15 | Side | OH | 21.8 |
| ARG | 3 | Side | NH2 | TYR | 15 | Side | OH | 10.4 |
| ASN | 5 | Side | ND2 | TYS | 14 | Side | OS4 | 62.5 |
| ASN | 5 | Side | ND2 | TYS | 14 | Side | OS2 | 21.9 |
| ASN | 7 | Side | ND2 | ASP | 2 | Main | O | 55.5 |
| ASN | 7 | Side | ND2 | GLN | 4 | Side | NE2 | 31.9 |
| ASN | 7 | Side | OD1 | GLN | 4 | Side | NE2 | 25.4 |
| ASN | 7 | Side | ND2 | GLN | 4 | Side | OE1 | 22.6 |
| THR | 8 | Main | O | SER | 17 | Side | OG | 66.1 |
| THR | 8 | Side | OG1 | GLN | 21 | Side | NE2 | 23.7 |
| ARG | 9 | Side | NH2 | GLN | 4 | Side | NE2 | 32.9 |
| ARG | 9 | Side | NE | GLN | 4 | Side | NE2 | 10.7 |
| ARG | 9 | Side | NH2 | ASP | 11 | Main | O | 40.7 |
| ARG | 9 | Side | NH1 | ASP | 11 | Main | O | 25.5 |
| ARG | 9 | Side | NH1 | ASP | 11 | Side | OD1 | 69.6 |
| ARG | 9 | Side | NH2 | ASP | 11 | Side | OD1 | 49.8 |
| ARG | 9 | Side | NH1 | ASP | 11 | Side | OD2 | 47.4 |
| ARG | 9 | Side | NH2 | ASP | 11 | Side | OD2 | 31.1 |
| ARG | 9 | Side | NH1 | GLN | 188 | Side | NE2 | 39.9 |
| LYS | 10 | Main | N | SER | 17 | Side | OG | 12.5 |
| LYS | 10 | Main | O | GLN | 188 | Side | NE2 | 84.1 |
| LYS | 10 | Main | O | LYS | 191 | Side | NZ | 24.5 |
| LYS | 10 | Side | NZ | ASP | 11 | Side | OD1 | 14.3 |
| LYS | 10 | Side | NZ | ASP | 11 | Side | OD2 | 13.1 |
| LYS | 10 | Side | NZ | ASN | 13 | Main | O | 47 |
| LYS | 10 | Side | NZ | THR | 16 | Side | OG1 | 36.2 |
| LYS | 10 | Side | NZ | SER | 17 | Side | OG | 90.1 |
| ARG | 11 | Side | NH2 | GLU | 172 | Side | OE2 | 31.8 |
| ARG | 11 | Side | NH1 | PRO | 183 | Main | O | 53.3 |
| ARG | 11 | Side | NH2 | TYR | 184 | Side | OH | 32 |
| ARG | 11 | Side | NH1 | TYR | 184 | Side | OH | 9 |
| ARG | 11 | Side | NE | GLN | 188 | Side | OE1 | 59.2 |
| ARG | 11 | Side | NH1 | GLN | 188 | Side | OE1 | 50.6 |
| VAL | 12 | Main | N | GLU | 262 | Side | OE2 | 21.2 |
| VAL | 12 | Main | N | GLU | 262 | Side | OE1 | 10 |
| SER | 13 | Side | OG | GLU | 172 | Side | OE1 | 30.5 |
| SER | 13 | Side | OG | GLU | 172 | Side | OE2 | 23.5 |
| SER | 13 | Side | OG | THR | 177 | Side | OG1 | 36.1 |
| SER | 13 | Side | OG | THR | 177 | Side | OG1 | 23.7 |
| SER | 13 | Side | OG | SER | 179 | Side | OG | 21 |
| LEU | 14 | Main | O | TYR | 89 | Side | OH | 99.9 |
| LEU | 14 | Main | N | CYS | 178 | Main | O | 70.6 |
| GLY | 17 | Main | N | TRP | 86 | Side | NE1 | 39.8 |
| GLY | 17 | Main | O | TYR | 108 | Side | OH | 98.6 |
| GLY | 17 | Main | O | TYR | 251 | Side | OH | 10.8 |
| ARG | 18 | Main | N | TYR | 108 | Side | OH | 9.6 |
| ARG | 18 | Main | N | GLU | 283 | Side | OE1 | 73.9 |
| ARG | 18 | Main | N | GLU | 283 | Side | OE2 | 25.7 |
| ARG | 18 | Side | NE | PHE | 247 | Main | O | 93 |
| ARG | 18 | Side | NH2 | PHE | 247 | Main | O | 65.6 |
| ARG | 18 | Side | NH1 | GLU | 283 | Side | OE1 | 98.3 |
| ARG | 18 | Side | NH1 | GLU | 283 | Side | OE2 | 21.7 |
| ARG | 18 | Side | NH2 | GLY | 286 | Main | O | 20.9 |
| ARG | 18 | Side | NH2 | HIS | 289 | Side | NE2 | 100 |
| TRP | 20 | Main | N | MET | 279 | Side | SD | 28.7 |
| TRP | 20 | Side | NE1 | TYR | 251 | Side | OH | 88 |
| TYR | 21 | Side | OH | LYS | 26 | Side | NZ | 37.5 |
| THR | 22 | Main | N | ASP | 276 | Side | OD2 | 88.7 |
| THR | 22 | Main | N | ASP | 276 | Side | OD1 | 88.2 |
| THR | 22 | Side | OG1 | ASP | 276 | Side | OD2 | 94 |
| THR | 22 | Side | OG1 | ASP | 276 | Side | OD1 | 46.9 |
| THR | 23 | Side | OG1 | GLU | 172 | Side | OE2 | 32.5 |
| GLY | 24 | Main | N | CYS | 20 | Main | O | 15.7 |
| GLY | 24 | Main | N | GLN | 21 | Main | O | 80.7 |
| GLY | 24 | Main | O | GLY | 173 | Main | N | 70.9 |
| GLN | 25 | Side | NE2 | LYS | 171 | Main | O | 16.6 |
| GLN | 25 | Side | NE2 | TYR | 184 | Side | OH | 62.4 |
| ASP | 29 | Main | O | ASP | 2 | Main | N | 60.2 |
| ASP | 29 | Main | N | ASP | 2 | Side | OD1 | 21.6 |
| ASP | 29 | Main | N | ASP | 2 | Side | OD2 | 18.6 |
| ARG | 31 | Main | N | TYS | 3 | Side | OS2 | 71.4 |
| ARG | 31 | Main | N | TYS | 3 | Side | OS3 | 26.9 |
| ARG | 31 | Side | NH2 | TYS | 3 | Side | OS4 | 74.1 |
| ARG | 31 | Side | NE | TYS | 3 | Side | OS4 | 71.9 |
| ARG | 31 | Side | NE | TYS | 3 | Side | OS2 | 22.5 |
| ARG | 31 | Side | NH2 | TYS | 3 | Side | OS2 | 21.1 |
| ARG | 31 | Side | NH2 | TYS | 3 | Side | OS3 | 14.6 |
| LYS | 32 | Main | N | TYS | 14 | Side | OS2 | 76.1 |
| LYS | 32 | Side | NZ | GLN | 4 | Main | O | 11.9 |
| LYS | 32 | Side | NZ | GLN | 4 | Side | OE1 | 29.9 |
| LYS | 32 | Side | NZ | ASN | 13 | Side | OD1 | 15.3 |
| LYS | 32 | Side | NZ | TYR | 15 | Side | OH | 19.1 |
| ALA | 33 | Main | N | TYS | 14 | Side | OS2 | 93.7 |
| ALA | 33 | Main | N | TYS | 14 | Side | OS3 | 26.7 |

Complex 3:

| V3 loop Residue | Number | Group | Atom | CCR5 Residue | Number | Group | Atom | Occupancy (%) |
| --- | --- | --- | --- | --- | --- | --- | --- | --- |
| ARG | 3 | Main | N | TYS | 14 | Side | OS3 | 41.96 |
| ARG | 3 | Main | N | TYS | 14 | Side | OS4 | 18.98 |
| ARG | 3 | Side | NE | TYS | 14 | Side | OS2 | 52.75 |
| ARG | 3 | Side | NH1 | TYS | 14 | Side | OS2 | 47.65 |
| ARG | 3 | Side | NE | TYS | 14 | Side | OS3 | 26.47 |
| ARG | 3 | Side | NH1 | TYS | 14 | Side | OS3 | 22.78 |
| ARG | 3 | Side | NE | TYS | 14 | Side | OS4 | 16.78 |
| ARG | 3 | Side | NH1 | TYS | 14 | Side | OS4 | 14.89 |
| ASN | 5 | Side | ND2 | TYS | 14 | Side | OS4 | 14.69 |
| ASN | 5 | Side | ND2 | TYS | 14 | Side | OS2 | 10.39 |
| ASN | 5 | Side | ND2 | TYS | 14 | Side | OS3 | 8.89 |
| ASN | 5 | Side | ND2 | TYR | 15 | Side | OH | 10.89 |
| ASN | 6 | Side | ND2 | GLU | 18 | Side | OE1 | 15.68 |
| ASN | 6 | Side | ND2 | GLU | 18 | Side | OE2 | 13.89 |
| ASN | 7 | Side | ND2 | GLN | 4 | Side | NE2 | 17.18 |
| ASN | 7 | Side | ND2 | GLN | 4 | Side | NE2 | 14.89 |
| THR | 8 | Main | O | SER | 17 | Side | OG | 46.85 |
| THR | 8 | Side | OG1 | GLN | 21 | Side | NE2 | 38.26 |
| ARG | 9 | Side | NH1 | GLN | 4 | Side | OE1 | 33.77 |
| ARG | 9 | Side | NH1 | SER | 6 | Side | OG | 48.65 |
| ARG | 9 | Side | NH2 | ASP | 11 | Side | OD2 | 56.84 |
| ARG | 9 | Side | NH1 | ASP | 11 | Side | OD2 | 54.95 |
| ARG | 9 | Side | NH2 | ASP | 11 | Side | OD1 | 41.46 |
| ARG | 9 | Side | NH1 | ASP | 11 | Side | OD1 | 37.76 |
| ARG | 9 | Side | NH1 | GLN | 188 | Side | NE2 | 21.08 |
| LYS | 10 | Main | O | GLN | 188 | Side | NE2 | 46.35 |
| LYS | 10 | Side | NZ | ASP | 11 | Side | OD1 | 52.95 |
| LYS | 10 | Side | NZ | ASP | 11 | Side | OD2 | 35.46 |
| LYS | 10 | Side | NZ | SER | 17 | Side | OG | 91.81 |
| LYS | 10 | Side | NZ | GLN | 188 | Side | OE1 | 9.89 |
| ARG | 11 | Side | NH1 | GLN | 170 | Side | NE2 | 11.09 |
| ARG | 11 | Side | NH2 | GLU | 172 | Side | OE1 | 62.64 |
| ARG | 11 | Side | NH1 | GLU | 172 | Side | OE1 | 49.95 |
| ARG | 11 | Side | NH2 | GLU | 172 | Side | OE2 | 31.77 |
| ARG | 11 | Side | NH1 | GLU | 172 | Side | OE2 | 10.19 |
| ARG | 11 | Side | NE | SER | 179 | Side | OG | 21.28 |
| ARG | 11 | Side | NH1 | TYR | 184 | Side | OH | 51.85 |
| ARG | 11 | Side | NE | TYR | 184 | Side | OH | 38.66 |
| VAL | 12 | Main | O | SER | 179 | Side | OG | 10.09 |
| VAL | 12 | Main | O | SER | 180 | Main | N | 30.47 |
| VAL | 12 | Main | N | SER | 180 | Side | OG | 20.68 |
| VAL | 12 | Main | O | SER | 180 | Side | OG | 31.07 |
| SER | 13 | Side | OG | GLU | 172 | Side | OE1 | 26.87 |
| SER | 13 | Side | OG | SER | 179 | Side | OG | 35.76 |
| LEU | 14 | Main | O | TYR | 89 | Side | OH | 95.8 |
| LEU | 14 | Main | N | CYS | 178 | Main | O | 97.6 |
| GLY | 15 | Main | O | TYR | 89 | Side | OH | 37.96 |
| ARG | 18 | Main | N | TYR | 37 | Side | OH | 40.26 |
| ARG | 18 | Main | O | TYR | 251 | Side | OH | 99.8 |
| ARG | 18 | Main | N | GLU | 283 | Side | OE2 | 58.14 |
| ARG | 18 | Main | N | GLU | 283 | Side | OE1 | 40.66 |
| ARG | 18 | Side | NE | TYR | 108 | Side | OH | 99.5 |
| ARG | 18 | Side | NH2 | TYR | 108 | Side | OH | 59.34 |
| ARG | 18 | Side | NH1 | GLU | 283 | Main | O | 58.34 |
| ARG | 18 | Side | NH1 | GLU | 283 | Side | OE1 | 60.14 |
| ARG | 18 | Side | NH1 | GLU | 283 | Side | OE2 | 42.96 |
| ARG | 18 | Side | NH2 | GLY | 286 | Main | O | 99.8 |
| ARG | 18 | Side | NH1 | GLY | 286 | Main | O | 76.12 |
| ARG | 18 | Side | NH2 | HIS | 289 | Side | NE2 | 12.29 |
| VAL | 19 | Main | N | GLU | 283 | Side | OE2 | 13.29 |
| TRP | 20 | Main | N | MET | 279 | Side | SD | 41.56 |
| TRP | 20 | Side | NE1 | TYR | 251 | Side | OH | 68.63 |
| TYR | 21 | Side | OH | ASN | 24 | Main | O | 56.74 |
| TYR | 21 | Side | OH | ASN | 24 | Side | ND2 | 31.67 |
| TYR | 21 | Side | OH | VAL | 25 | Main | O | 24.28 |
| TYR | 21 | Side | OH | GLN | 280 | Side | NE2 | 77.02 |
| THR | 22 | Main | N | ASP | 276 | Side | OD1 | 86.61 |
| THR | 22 | Main | N | ASP | 276 | Side | OD2 | 77.62 |
| THR | 22 | Side | OG1 | ASN | 258 | Side | ND2 | 11.59 |
| THR | 22 | Side | OG1 | GLU | 262 | Side | OE1 | 48.85 |
| THR | 22 | Side | OG1 | GLU | 262 | Side | OE2 | 18.58 |
| THR | 22 | Side | OG1 | ASP | 276 | Side | OD1 | 16.38 |
| THR | 23 | Side | OG1 | GLU | 172 | Side | OE2 | 29.27 |
| THR | 23 | Side | OG1 | GLU | 172 | Side | OE1 | 11.79 |
| GLY | 24 | Main | N | CYS | 20 | Main | O | 17.18 |
| GLY | 24 | Main | N | GLN | 21 | Main | O | 14.69 |
| GLN | 25 | Side | OE1 | MET | 1 | Main | N | 54.15 |
| ILE | 26 | Main | N | GLN | 21 | Side | OE1 | 11.29 |
| ARG | 31 | Main | N | TYS | 3 | Side | OS4 | 44.06 |
| ARG | 31 | Main | N | TYS | 3 | Side | OS2 | 35.06 |
| ARG | 31 | Main | N | TYS | 3 | Side | OS3 | 33.47 |
| ARG | 31 | Side | NE | TYS | 3 | Side | OS3 | 41.76 |
| ARG | 31 | Side | NE | TYS | 3 | Side | OS2 | 38.16 |
| ARG | 31 | Side | NH2 | TYS | 3 | Side | OS3 | 30.77 |
| ARG | 31 | Side | NH2 | TYS | 3 | Side | OS2 | 29.97 |
| ARG | 31 | Side | NE | TYS | 3 | Side | OS4 | 28.67 |
| ARG | 31 | Side | NH2 | TYS | 3 | Side | OS4 | 21.98 |
| LYS | 32 | Side | NZ | SER | 6 | Main | O | 88.61 |
| LYS | 32 | Side | NZ | SER | 6 | Side | OG | 88.41 |
| LYS | 32 | Side | NZ | ASP | 11 | Main | O | 10.39 |
| LYS | 32 | Side | NZ | ASP | 11 | Side | OD2 | 31.27 |
| LYS | 32 | Side | NZ | ASP | 11 | Side | OD1 | 24.18 |
| ALA | 33 | Main | N | TYS | 14 | Side | OS4 | 39.56 |
| ALA | 33 | Main | N | TYS | 14 | Side | OS2 | 18.18 |
| ALA | 33 | Main | N | TYS | 14 | Side | OS3 | 16.18 |
| HIS | 34 | Main | N | TYS | 14 | Side | OS4 | 17.28 |

Complex 6:

| V3 loop Residue | Number | Group | Atom | CCR5 Residue | Number | Group | Atom | Occupancy (%) |
| --- | --- | --- | --- | --- | --- | --- | --- | --- |
| ARG | 3 | Side | NH2 | ASP | 11 | Side | OD1 | 35.3 |
| ASN | 5 | Side | ND2 | ASP | 11 | Main | O | 18.5 |
| ASN | 5 | Side | ND2 | ILE | 12 | Main | O | 9.1 |
| ASN | 5 | Side | ND2 | TYS | 14 | Side | OS2 | 16.8 |
| ASN | 5 | Side | ND2 | TYS | 14 | Side | OS3 | 16.2 |
| ASN | 5 | Side | ND2 | TYS | 14 | Side | OS4 | 15.8 |
| ASN | 5 | Side | ND2 | TYS | 14 | Side | OH | 11.3 |
| ASN | 6 | Main | N | TYS | 14 | Side | OS3 | 36.1 |
| ASN | 6 | Main | N | TYS | 14 | Side | OS2 | 33.6 |
| ASN | 6 | Main | N | TYS | 14 | Side | OS4 | 29.9 |
| ASN | 6 | Side | ND2 | TYS | 14 | Side | OS3 | 23.7 |
| ASN | 6 | Side | ND2 | TYS | 14 | Side | OS2 | 22.9 |
| ASN | 6 | Side | ND2 | TYS | 14 | Side | OS4 | 21.5 |
| ASN | 7 | Side | ND2 | ASP | 2 | Side | OD2 | 11.8 |
| ASN | 7 | Side | ND2 | TYS | 3 | Side | OS4 | 25.1 |
| ASN | 7 | Side | ND2 | TYS | 3 | Side | OS3 | 12.2 |
| ASN | 7 | Side | ND2 | GLN | 4 | Side | OE1 | 18.9 |
| ASN | 7 | Side | OD1 | GLN | 4 | Side | NE2 | 12.9 |
| ASN | 7 | Side | ND2 | ASP | 11 | Side | OD2 | 15.5 |
| THR | 8 | Main | O | GLN | 4 | Side | NE2 | 26.2 |
| THR | 8 | Main | N | ASP | 11 | Side | OD2 | 60.9 |
| THR | 8 | Main | N | ASP | 11 | Side | OD1 | 41.1 |
| THR | 8 | Main | O | GLN | 188 | Side | NE2 | 10.7 |
| THR | 8 | Side | OG1 | TYS | 10 | Main | O | 9.2 |
| THR | 8 | Side | OG1 | ASP | 11 | Side | OD2 | 24.1 |
| THR | 8 | Side | OG1 | ASP | 11 | Side | OD1 | 14.9 |
| THR | 8 | Side | OG1 | SER | 17 | Side | OG | 11.6 |
| ARG | 9 | Side | NH2 | ASP | 2 | Side | OD1 | 84.7 |
| ARG | 9 | Side | NH1 | ASP | 2 | Side | OD1 | 84.1 |
| ARG | 9 | Side | NH1 | ASP | 2 | Side | OD2 | 28.9 |
| ARG | 9 | Side | NH2 | ASP | 2 | Side | OD2 | 16.2 |
| ARG | 9 | Side | NH1 | GLN | 4 | Side | OE1 | 47.3 |
| ARG | 9 | Side | NH1 | GLN | 4 | Side | NE2 | 10.5 |
| ARG | 9 | Side | NH1 | TYS | 10 | Side | OS2 | 22 |
| ARG | 9 | Side | NH2 | PRO | 183 | Main | O | 94.1 |
| ARG | 9 | Side | NE | PRO | 183 | Main | O | 67 |
| ARG | 9 | Side | NH1 | GLN | 188 | Side | NE2 | 10.7 |
| LYS | 10 | Main | N | TYS | 10 | Side | OS3 | 32.5 |
| LYS | 10 | Main | N | TYS | 10 | Side | OS4 | 11.5 |
| LYS | 10 | Main | N | TYS | 10 | Side | OH | 10 |
| LYS | 10 | Main | O | GLN | 188 | Side | NE2 | 20 |
| LYS | 10 | Main | N | GLN | 188 | Side | OE1 | 14.6 |
| LYS | 10 | Main | O | LYS | 191 | Side | NZ | 34.8 |
| LYS | 10 | Side | NZ | GLN | 261 | Side | OE1 | 82.2 |
| LYS | 10 | Side | NZ | GLU | 262 | Side | OE1 | 98.8 |
| LYS | 10 | Side | NZ | SER | 272 | Side | OG | 76.3 |
| ARG | 11 | Side | NH1 | GLU | 172 | Main | O | 85.9 |
| ARG | 11 | Side | NH2 | GLU | 172 | Main | O | 36.2 |
| ARG | 11 | Side | NH2 | THR | 177 | Side | OG1 | 66.1 |
| ARG | 11 | Side | NH1 | THR | 177 | Side | OG1 | 16.7 |
| ARG | 11 | Side | NH2 | SER | 179 | Side | OG | 70 |
| VAL | 12 | Main | O | SER | 179 | Side | OG | 22.1 |
| VAL | 12 | Main | O | SER | 180 | Main | N | 51.2 |
| VAL | 12 | Main | O | SER | 180 | Side | OG | 10.5 |
| SER | 13 | Side | OG | THR | 177 | Side | OG1 | 33 |
| SER | 13 | Side | OG | SER | 179 | Side | OG | 48.3 |
| LEU | 14 | Main | O | TYR | 89 | Side | OH | 99.6 |
| LEU | 14 | Main | N | CYS | 178 | Main | O | 99.2 |
| ARG | 18 | Main | O | TYR | 251 | Side | OH | 100 |
| ARG | 18 | Main | N | GLU | 283 | Side | OE1 | 57.5 |
| ARG | 18 | Main | N | GLU | 283 | Side | OE2 | 43 |
| ARG | 18 | Side | NE | TYR | 37 | Side | OH | 53.4 |
| ARG | 18 | Side | NH1 | GLY | 111 | Main | O | 20.5 |
| ARG | 18 | Side | NE | GLU | 283 | Side | OE1 | 98.9 |
| ARG | 18 | Side | NE | GLU | 283 | Side | OE2 | 98 |
| ARG | 18 | Side | NH2 | GLU | 283 | Side | OE2 | 59.8 |
| ARG | 18 | Side | NH2 | GLU | 283 | Side | OE1 | 45.5 |
| ARG | 18 | Side | NH2 | GLY | 286 | Main | O | 58.5 |
| ARG | 18 | Side | NH2 | MET | 287 | Main | N | 21.4 |
| ARG | 18 | Side | NH1 | HIS | 289 | Side | NE2 | 99.9 |
| ARG | 18 | Side | NH2 | HIS | 289 | Side | NE2 | 30.8 |
| TRP | 20 | Main | N | MET | 279 | Side | SD | 30.2 |
| TRP | 20 | Side | NE1 | TYR | 108 | Side | OH | 91.9 |
| TYR | 21 | Side | OH | ASN | 24 | Main | O | 9.2 |
| TYR | 21 | Side | OH | LYS | 26 | Side | NZ | 11.4 |
| TYR | 21 | Side | OH | GLN | 280 | Side | NE2 | 23.5 |
| THR | 22 | Main | N | ASP | 276 | Side | OD2 | 94.8 |
| THR | 22 | Main | N | ASP | 276 | Side | OD1 | 69.5 |
| THR | 22 | Side | OG1 | SER | 272 | Main | O | 15.6 |
| THR | 22 | Side | OG1 | SER | 272 | Side | OG | 85.7 |
| THR | 22 | Side | OG1 | ASP | 276 | Side | OD1 | 84 |
| THR | 22 | Side | OG1 | ASP | 276 | Side | OD2 | 52.8 |
| GLY | 24 | Main | N | CYS | 20 | Main | O | 95.1 |
| GLY | 24 | Main | N | CYS | 20 | Side | SG | 12.2 |
| GLN | 25 | Side | NE2 | GLU | 172 | Side | OE2 | 11.2 |
| ILE | 26 | Main | N | GLU | 172 | Side | OE1 | 18 |
| ASP | 29 | Side | OD1 | MET | 1 | Main | N | 14.6 |
| ASP | 29 | Side | OD2 | MET | 1 | Main | N | 13.8 |
| ARG | 31 | Side | NH2 | GLN | 4 | Main | O | 87.8 |
| ARG | 31 | Side | NH1 | GLN | 4 | Main | O | 58.1 |
| ARG | 31 | Side | NH1 | GLN | 4 | Side | OE1 | 32.5 |
| ARG | 31 | Side | NH1 | GLN | 4 | Side | NE2 | 28.8 |
| ARG | 31 | Side | NH1 | ASP | 11 | Side | OD2 | 27.2 |
| ARG | 31 | Side | NH1 | ASP | 11 | Side | OD1 | 16.3 |
| LYS | 32 | Main | N | TYS | 3 | Side | OH | 32.7 |
| LYS | 32 | Side | NZ | TYS | 3 | Side | OS3 | 42.2 |
| LYS | 32 | Side | NZ | TYS | 3 | Side | OS2 | 25.9 |
| LYS | 32 | Side | NZ | TYS | 3 | Side | OS4 | 19.6 |
| LYS | 32 | Side | NZ | TYS | 3 | Side | OH | 12.6 |
| HIS | 34 | Side | ND1 | TYS | 3 | Main | O | 10.2 |

Complex 12:

| V3 loop Residue | Number | Group | Atom | CCR5 Residue | Number | Group | Atom | Occupancy (%) |
| --- | --- | --- | --- | --- | --- | --- | --- | --- |
| ARG | 3 | Side | NH1 | GLU | 18 | Side | OE2 | 85 |
| ARG | 3 | Side | NH2 | GLU | 18 | Side | OE1 | 81.2 |
| ARG | 3 | Side | NH1 | GLU | 18 | Side | OE1 | 60.5 |
| ARG | 3 | Side | NH2 | GLU | 18 | Side | OE2 | 45.3 |
| ASN | 5 | Side | OD1 | TYS | 14 | Main | N | 80.4 |
| ASN | 5 | Side | OD1 | TYR | 15 | Main | N | 85.7 |
| ASN | 7 | Side | ND2 | TYS | 3 | Side | OH | 63.7 |
| THR | 8 | Main | O | GLN | 4 | Side | NE2 | 34.7 |
| THR | 8 | Main | N | TYS | 14 | Side | OS2 | 77.5 |
| THR | 8 | Main | N | TYS | 14 | Side | OS3 | 13.2 |
| ARG | 9 | Side | NH2 | ASP | 2 | Side | OD1 | 70.3 |
| ARG | 9 | Side | NH2 | ASP | 2 | Side | OD2 | 69.9 |
| ARG | 9 | Side | NH1 | ASP | 2 | Side | OD1 | 69.2 |
| ARG | 9 | Side | NH1 | ASP | 2 | Side | OD2 | 65.3 |
| ARG | 9 | Side | NH2 | TYS | 3 | Main | O | 20 |
| ARG | 9 | Side | NE | GLN | 4 | Side | OE1 | 9 |
| ARG | 9 | Side | NH2 | PRO | 183 | Main | O | 90.5 |
| ARG | 9 | Side | NE | PRO | 183 | Main | O | 53.9 |
| LYS | 10 | Main | N | GLN | 4 | Side | OE1 | 13.4 |
| LYS | 10 | Main | O | LYS | 191 | Side | NZ | 42.2 |
| LYS | 10 | Side | NZ | SER | 17 | Side | OG | 60.6 |
| LYS | 10 | Side | NZ | GLN | 261 | Main | O | 21.3 |
| LYS | 10 | Side | NZ | GLN | 261 | Side | OE1 | 50.8 |
| LYS | 10 | Side | NZ | GLU | 262 | Side | OE2 | 18 |
| ARG | 11 | Side | NH2 | GLN | 170 | Side | NE2 | 15.9 |
| ARG | 11 | Side | NH1 | LYS | 171 | Main | O | 38.9 |
| ARG | 11 | Side | NH1 | GLU | 172 | Side | OE2 | 93.3 |
| ARG | 11 | Side | NE | GLU | 172 | Side | OE2 | 50.9 |
| ARG | 11 | Side | NE | SER | 179 | Side | OG | 52 |
| ARG | 11 | Side | NH2 | TYR | 184 | Side | OH | 89.7 |
| VAL | 12 | Main | O | SER | 179 | Side | OG | 21.2 |
| VAL | 12 | Main | O | SER | 180 | Main | N | 68.9 |
| SER | 13 | Side | OG | GLU | 172 | Side | OE2 | 94.9 |
| SER | 13 | Side | OG | GLU | 172 | Side | OE1 | 87.9 |
| SER | 13 | Side | OG | THR | 177 | Side | OG1 | 15.2 |
| SER | 13 | Side | OG | SER | 179 | Side | OG | 56.1 |
| LEU | 14 | Main | O | TYR | 89 | Side | OH | 98.3 |
| LEU | 14 | Main | N | CYS | 178 | Main | O | 99.8 |
| GLY | 15 | Main | O | TYR | 89 | Side | OH | 41.1 |
| GLY | 17 | Main | N | ALA | 90 | Main | N | 22.6 |
| ARG | 18 | Main | N | TYR | 37 | Side | OH | 80.8 |
| ARG | 18 | Main | O | TYR | 251 | Side | OH | 99.8 |
| ARG | 18 | Main | N | GLU | 283 | Side | OE1 | 16.3 |
| ARG | 18 | Side | NH2 | TYR | 108 | Side | OH | 34.9 |
| ARG | 18 | Side | NE | GLU | 283 | Side | OE2 | 99.5 |
| ARG | 18 | Side | NE | GLU | 283 | Side | OE1 | 94.7 |
| ARG | 18 | Side | NH1 | GLU | 283 | Side | OE1 | 77.7 |
| ARG | 18 | Side | NH1 | GLU | 283 | Side | OE2 | 72.5 |
| ARG | 18 | Side | NH1 | GLY | 286 | Main | O | 88.3 |
| ARG | 18 | Side | NH2 | HIS | 289 | Side | NE2 | 97.8 |
| ARG | 18 | Side | NH1 | HIS | 289 | Side | NE2 | 26.8 |
| TRP | 20 | Main | N | MET | 279 | Side | SD | 36.5 |
| TRP | 20 | Side | NE1 | TYR | 251 | Side | OH | 77 |
| TYR | 21 | Side | OH | ASN | 24 | Main | O | 77.3 |
| TYR | 21 | Side | OH | ASN | 24 | Side | ND2 | 37.2 |
| TYR | 21 | Side | OH | VAL | 25 | Main | O | 18.5 |
| TYR | 21 | Side | OH | GLN | 280 | Side | NE2 | 83.5 |
| THR | 22 | Main | N | ASP | 276 | Side | OD2 | 9.2 |
| THR | 22 | Side | OG1 | GLU | 262 | Side | OE2 | 84.2 |
| THR | 23 | Side | OG1 | GLU | 172 | Side | OE2 | 87.3 |
| THR | 23 | Side | OG1 | GLU | 172 | Side | OE1 | 72.4 |
| GLY | 24 | Main | N | CYS | 20 | Main | O | 95.1 |
| ILE | 26 | Main | N | GLN | 21 | Side | OE1 | 80.4 |
| GLY | 28 | Main | N | TYS | 3 | Side | OS4 | 18.4 |
| GLY | 28 | Main | N | TYS | 3 | Side | OS3 | 14.1 |
| ASP | 29 | Main | N | TYS | 3 | Side | OS4 | 40 |
| ASP | 29 | Main | N | TYS | 3 | Side | OS3 | 27.8 |
| ASP | 29 | Main | N | TYS | 3 | Side | OS2 | 12.9 |
| ARG | 31 | Side | NH2 | ASP | 11 | Main | O | 15.1 |
| ARG | 31 | Side | NH1 | ASP | 11 | Side | OD2 | 15.2 |
| ARG | 31 | Side | NH2 | ASP | 11 | Side | OD2 | 10 |
| ARG | 31 | Side | NH2 | TYS | 14 | Side | OS4 | 75.2 |
| ARG | 31 | Side | NE | TYS | 14 | Side | OS4 | 56 |
| ARG | 31 | Side | NH2 | TYS | 14 | Side | OS3 | 12 |
| LYS | 32 | Side | NZ | ASN | 13 | Side | OD1 | 73 |

Complex 14:

| V3 loop Residue | Number | Group | Atom | CCR5 Residue | Number | Group | Atom | Occupancy (%) |
| --- | --- | --- | --- | --- | --- | --- | --- | --- |
| ARG | 3 | Side | NH2 | ILE | 12 | Main | O | 12.9 |
| ARG | 3 | Side | NH2 | TYR | 15 | Side | OH | 44.6 |
| ASN | 5 | Side | ND2 | ASP | 11 | Main | O | 18.9 |
| ASN | 5 | Side | ND2 | ILE | 12 | Main | O | 17.3 |
| ASN | 5 | Side | OD1 | TYS | 14 | Main | N | 50.4 |
| ASN | 5 | Side | OD1 | TYR | 15 | Main | N | 61.6 |
| ASN | 6 | Main | N | GLU | 18 | Side | OE2 | 58.6 |
| ASN | 6 | Side | ND2 | GLU | 18 | Side | OE2 | 28.5 |
| ASN | 6 | Side | ND2 | GLU | 18 | Side | OE1 | 25.4 |
| ASN | 7 | Side | OD1 | GLN | 4 | Main | N | 18.3 |
| ASN | 7 | Side | ND2 | TYS | 14 | Side | OS4 | 10.6 |
| THR | 8 | Main | N | TYS | 14 | Side | OS4 | 90.8 |
| THR | 8 | Side | OG1 | GLU | 18 | Main | O | 34.4 |
| ARG | 9 | Side | NH2 | ASP | 2 | Side | OD1 | 59.1 |
| ARG | 9 | Side | NH2 | ASP | 2 | Side | OD2 | 44.3 |
| ARG | 9 | Side | NH1 | ASP | 2 | Side | OD1 | 36.3 |
| ARG | 9 | Side | NH1 | ASP | 2 | Side | OD2 | 24.1 |
| ARG | 9 | Side | NE | GLN | 4 | Side | NE2 | 30.4 |
| ARG | 9 | Side | NE | GLN | 4 | Side | OE1 | 10.2 |
| ARG | 9 | Side | NH2 | GLN | 4 | Side | OE1 | 9.7 |
| ARG | 9 | Side | NE | PRO | 183 | Main | O | 92.4 |
| ARG | 9 | Side | NH2 | PRO | 183 | Main | O | 67.9 |
| ARG | 9 | Side | NH2 | TYR | 184 | Main | O | 15 |
| LYS | 10 | Main | N | GLN | 188 | Side | OE1 | 82.5 |
| LYS | 10 | Main | O | LYS | 191 | Side | NZ | 86.1 |
| LYS | 10 | Side | NZ | SER | 17 | Main | O | 25.9 |
| LYS | 10 | Side | NZ | SER | 17 | Side | OG | 50.2 |
| LYS | 10 | Side | NZ | GLN | 261 | Main | O | 30.4 |
| LYS | 10 | Side | NZ | SER | 272 | Side | OG | 14 |
| ARG | 11 | Side | NH1 | GLN | 170 | Side | OE1 | 63.9 |
| ARG | 11 | Side | NE | GLN | 170 | Side | OE1 | 40.5 |
| ARG | 11 | Side | NE | GLN | 170 | Side | NE2 | 21.1 |
| ARG | 11 | Side | NH1 | GLN | 170 | Side | NE2 | 19.9 |
| ARG | 11 | Side | NE | GLU | 172 | Side | OE1 | 11.6 |
| ARG | 11 | Side | NH1 | GLU | 172 | Side | OE1 | 10.5 |
| ARG | 11 | Side | NH1 | SER | 179 | Side | OG | 32.9 |
| ARG | 11 | Side | NH2 | SER | 179 | Side | OG | 9.1 |
| ARG | 11 | Side | NH2 | SER | 180 | Main | O | 99.7 |
| ARG | 11 | Side | NH1 | SER | 180 | Main | O | 98.2 |
| ARG | 11 | Side | NH1 | HIS | 181 | Side | ND1 | 10.4 |
| ARG | 11 | Side | NE | TYR | 184 | Side | OH | 55 |
| VAL | 12 | Main | N | SER | 179 | Side | OG | 88.6 |
| SER | 13 | Main | N | SER | 179 | Side | OG | 92 |
| SER | 13 | Side | OG | GLU | 172 | Main | O | 24.3 |
| SER | 13 | Side | OG | THR | 177 | Side | OG1 | 90.9 |
| LEU | 14 | Main | O | TYR | 89 | Side | OH | 34.4 |
| LEU | 14 | Main | N | CYS | 178 | Main | O | 90.8 |
| GLY | 17 | Main | N | TRP | 86 | Main | O | 19.6 |
| GLY | 17 | Main | N | ALA | 90 | Main | N | 14.3 |
| ARG | 18 | Main | O | TYR | 251 | Side | OH | 96.9 |
| ARG | 18 | Main | N | GLU | 283 | Side | OE2 | 59.1 |
| ARG | 18 | Main | N | GLU | 283 | Side | OE1 | 42 |
| ARG | 18 | Side | NE | TYR | 37 | Side | OH | 52.8 |
| ARG | 18 | Side | NH1 | TYR | 108 | Side | OH | 12.7 |
| ARG | 18 | Side | NE | GLU | 283 | Side | OE2 | 93.2 |
| ARG | 18 | Side | NE | GLU | 283 | Side | OE1 | 93.1 |
| ARG | 18 | Side | NH2 | GLU | 283 | Side | OE1 | 58.9 |
| ARG | 18 | Side | NH2 | GLU | 283 | Side | OE2 | 42.5 |
| ARG | 18 | Side | NH2 | GLY | 286 | Main | O | 99.7 |
| ARG | 18 | Side | NH1 | HIS | 289 | Side | NE2 | 99 |
| TRP | 20 | Main | N | MET | 279 | Side | SD | 47.6 |
| TRP | 20 | Side | NE1 | TYR | 251 | Side | OH | 24.7 |
| TYR | 21 | Side | OH | ILE | 23 | Main | O | 60.6 |
| TYR | 21 | Side | OH | VAL | 25 | Main | N | 55.3 |
| THR | 22 | Main | O | LYS | 22 | Side | NZ | 97.6 |
| THR | 22 | Main | N | ASP | 276 | Side | OD2 | 79.3 |
| THR | 22 | Main | N | ASP | 276 | Side | OD1 | 76 |
| THR | 22 | Side | OG1 | ASP | 276 | Side | OD1 | 74.9 |
| THR | 22 | Side | OG1 | ASP | 276 | Side | OD2 | 40.7 |
| THR | 23 | Side | OG1 | LYS | 22 | Main | N | 73.3 |
| THR | 23 | Side | OG1 | LYS | 22 | Main | O | 54.3 |
| GLY | 24 | Main | N | CYS | 20 | Main | O | 52.5 |
| GLY | 24 | Main | O | GLN | 21 | Side | NE2 | 71.4 |
| GLN | 25 | Side | NE2 | GLU | 172 | Side | OE1 | 40 |
| GLN | 25 | Side | NE2 | GLU | 172 | Side | OE2 | 16.3 |
| GLN | 25 | Side | OE1 | TYR | 184 | Side | OH | 64.5 |
| GLN | 25 | Side | NE2 | TYR | 184 | Side | OH | 31.3 |
| ASP | 29 | Side | OD1 | MET | 1 | Main | N | 99.3 |
| ASP | 29 | Side | OD2 | MET | 1 | Main | N | 73.1 |
| ASP | 29 | Side | OD2 | ASP | 2 | Main | N | 79.6 |
| ASP | 29 | Side | OD2 | TYS | 3 | Main | N | 91.1 |
| ILE | 30 | Main | N | TYS | 3 | Side | OS3 | 39.3 |
| ILE | 30 | Main | N | TYS | 3 | Side | OS4 | 27.4 |
| ILE | 30 | Main | N | TYS | 3 | Side | OS2 | 22.5 |
| ARG | 31 | Side | NH1 | GLN | 4 | Main | O | 96.3 |
| ARG | 31 | Side | NH1 | SER | 6 | Side | OG | 9.1 |
| ARG | 31 | Side | NH2 | ASP | 11 | Side | OD1 | 91.2 |
| ARG | 31 | Side | NE | ASP | 11 | Side | OD1 | 83.9 |
| ARG | 31 | Side | NE | ASP | 11 | Side | OD2 | 75.7 |
| ARG | 31 | Side | NH2 | ASP | 11 | Side | OD2 | 16 |
| ARG | 31 | Side | NH2 | TYS | 14 | Side | OS3 | 90.3 |
| ARG | 31 | Side | NH1 | TYS | 14 | Side | OS3 | 89.8 |
| LYS | 32 | Side | NZ | ASP | 11 | Side | OD2 | 63.2 |
